# Design Strategies for Coupling CO_2_ Reduction Molecular Electrocatalysts to Silicon Photocathodes

**DOI:** 10.1021/acsmaterialsau.5c00010

**Published:** 2025-04-14

**Authors:** Simran S. Saund, Melissa K. Gish, Jeremiah Choate, Trung H. Le, Smaranda C. Marinescu, Nathan R. Neale

**Affiliations:** † Chemistry and Nanoscience Center, National Renewable Energy Laboratory, Golden, Colorado 80401, United States; ‡ Department of Chemistry, University of Southern California, Los Angeles, California 90089, United States; § Renewable and Sustainable Energy Institute, University of Colorado Boulder, Boulder, Colorado 80309, United States

**Keywords:** solar fuels, hybrid photoelectrode, silicon, nanocrystal, carbon dioxide reduction, electrocatalyst

## Abstract

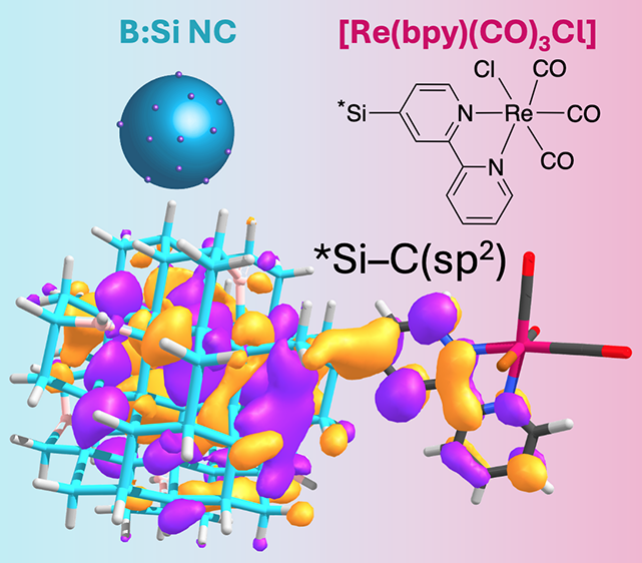

We explore strategies for enhancing the electronic interaction between silicon nanocrystals (Si NCs) and surface-tethered molecular Re electrocatalysts ([Re]) as models for CO_2_-reducing photocathodes. Using density functional theory (DFT) combined with electrochemical, spectroscopic, and photocatalytic measurements, we determine that the intrinsic Si (^i^Si) NC conduction band energy in ^i^Si–[Re] assemblies is below the [Re] lowest unoccupied molecular orbital (LUMO) and singly occupied molecular orbital energies even for strongly quantum-confined 3.0–3.9 nm diameter hydrogen- and methyl-terminated ^i^Si NCs, respectively. We computationally analyze design strategies to align the semiconductor conduction band edge and electrocatalyst frontier molecular orbitals by varying the ^i^Si NC size, introducing boron as a dopant in the Si NC, and modifying the attachment chemistry to the [Re] complex aryl ligand framework. Our DFT analysis identifies a target hybrid structure featuring B-doped silicon (B:Si) NCs and a direct bond between a surface atom and an sp^2^-hybridized carbon of the electrocatalyst bipyridine aryl ring ligand (B:Si–C_Ar_[Re]). We synthesize the B:Si–C_Ar_[Re] NC assembly and find evidence of direct hybridization between the B:Si NC and the surface [Re] electrocatalyst LUMO using electrochemical measurements and transient absorption spectroscopy. This work provides a blueprint for the design of new Si photocathode-molecular electrocatalyst hybrids for CO_2_ reduction and related fuel-forming photocatalytic conversions.

## Introduction

The storage of solar radiation in molecular bonds by photoreductive transformations is a long-sought-after goal in sustainable energy research.^1−3^ Combining efficient photon collection and conversion into chemical potential that is used in complex multistep reactions is vital for any photocatalytic system. Bulk silicon has long been the flagship choice for photovoltaic systems,^4^ and use of Si as a photocatalytic system has long been studied for photon to molecule energy conversion systems.^5−7^ However, the native surface chemistry of Si is ill-suited for catalytic turnover for either the carbon dioxide reduction reaction (CO_2_RR) or hydrogen evolution reaction (HER) and results in noncatalytic reactions at the Si surface.^5,8−13^ To avoid this challenge and take advantage of Si’s attractive light-harvesting properties and technological maturity, it has been suggested that tethering molecular electrocatalysts to Si photoelectrode surfaces may yield cheap, abundant, and efficient photoconversion systems.^14−16^

Recent work from several groups have explored semiconductor–molecular catalyst hybrids to independently tune light harvesting and catalysis in artificial photosynthesis schemes. While it has been noted that the absolute band positions of semiconductors are overemphasized in the design of photoelectrochemical systems, it is also clear that energetics play an important role in any system relying on a molecular (photo)catalyst with discrete molecular orbital energies.^17^ For example, Reisner and co-workers have shown the benefits of tethering a phosphonated Co(II) bis(terpyridine) molecular CO_2_RR catalyst to classical *z*-scheme doped metal oxide photocatalysts^18^ as well as biohybrid platforms such as cyanamide-functionalized carbon nitride-indium tin oxide-formate dehydrogenase.^19^ Other recent examples include the significant contributions of the CHASE Fuels from Sunlight Energy Innovation Hub that has provided new insights on electron transfer and other processes for a range of molecular CO_2_RR catalyst-semiconductor systems including Si photocathodes.^20−26^

Our lab recently introduced intrinsic silicon nanocrystal–molecular Re complex hybrid assemblies (Si–[Re] NCs) as model systems for understanding the energetic alignment of Si with prototypical CO_2_RR molecular electrocatalysts.^27^ This work concluded that the conduction band edge energy of 3.9 nm diameter Si NCs is likely misaligned with the lowest unoccupied molecular orbital (LUMO) of the tethered [Re] complexes and prevents productive energy cascade of light-driven Si conduction band electrons to [Re] LUMO orbitals that is essential for driving unassisted photocatalytic CO_2_RR. Consequently, we found photocatalytic CO_2_RR is dominated by direct light absorption from the surface-bound [Re] complexes. The results of our prior work begged the question: What are potential design strategies to achieve optimal energetic alignment in a hybrid silicon–CO_2_RR electrocatalyst artificial photosynthetic system?

In this article, we aim to answer this question by exploring several new strategies to realign the Si semiconductor conduction band edge energy with [Re] orbitals, including increasing quantum confinement in the intrinsic silicon (^i^Si) NCs, introducing boron as a dopant, and modifying the attachment chemistry to the [Re] complex aryl ligand framework. We study the effects of these strategies via density functional theory (DFT), cyclic voltammetry (CV), transient absorption spectroscopy (TAS), and photocatalytic CO_2_RR product analysis. First, we find that decreasing the Si NC size to increase the conduction band energy minimum (*E*_CB_) is a viable strategy but only for ultrasmall alkyl-terminated Si NCs with diameters < ∼2.0 nm and band gaps ≥ 2.0 eV. Second, we probe B-doped Si (B:Si) NCs and show that B-derived intragap states can electronically couple with the [Re] LUMO to generate hybridized B:Si–[Re] NC states. Third, we test the effect of the binding motif of the linker molecule tethering the molecular electrocatalyst to the B:Si NC surface and demonstrate that direct attachment of sp^2^-hydridized C atoms on the [Re] catalyst ligand framework to B:Si NC surface atoms affords a third viable strategy to generate a productive photocatalytic system.

## Results and Discussion

### Increasing Si *E*_CB_ via Quantum Confinement

We leverage plasma-enhanced chemical vapor deposition (PECVD) to grow 3.0, 3.3, 3.5, and 3.9 nm diameter ^i^Si NCs^28,29^ and use our previously reported [Re] complex attachment chemistry^27^ to assemble the Si–[Re] NC hybrid systems. Briefly, Si NCs are first undersaturated with dodecyl auxiliary ligands to provide stability and solubility. The product is then heated in the presence of [Re(mcabpy)(CO)_3_Br] ([Re]CHO, mcabpy = 4-methyl-2,2′-bipyridyl-4′-carboxaldehyde) yielding the product assembly, which we term ^i^Si–OCH_2_[Re] to distinguish from B:Si NCs and where different attachment chemistries are used (vide infra). The different species are characterized by diffuse reflectance infrared Fourier transform spectroscopy (DRIFTS, Figure S1). Qualitatively, the DRIFTS spectra for the series show a distinct trend in the ratio of C–H/Si–H peaks and Si–H/Re(CO)_3_ peaks. The C–H/Si–H ratio increases for smaller NCs likely due to size-dependent changes in surface morphology and the surface *SiH_*x*_ ratios (where *Si indicates a surface Si atom).^28−31^ The Re(CO)_3_/Si–H ratio also increases with decreasing NC diameter, and we observe a corresponding increase in back-bonded (O)*Si–H stretches. Lastly, the residual surface hydrides *SiH_*x*_ centered ∼2100 cm^–1^ are red-shifted for larger NC diameters, suggesting higher molecular coverage for larger NCs, consistent with quantitative FTIR experiments (vide infra). Expectedly, UV–vis (Figure S2) spectroscopy shows that the Si absorption curve blue shifts with decreasing NC size due to an increase in direct band gap character.^32^ The NC sizes are determined by comparing the photoluminescence (PL) maximum (Figure S2) to our previously reported sizing curve for dodecyl-bound intrinsic Si NC.^28^

**Figure 1:**
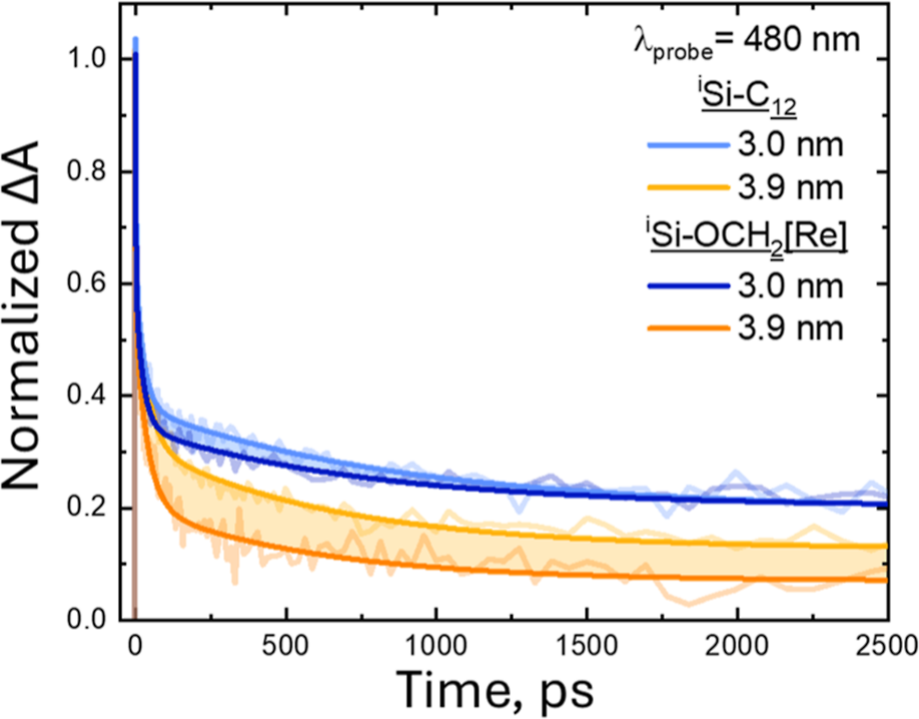
Normalized transient absorption dynamics at probe wavelengths of 480 nm after 400 nm photoexcitation for ^i^Si–C_12_ and ^i^Si–OCH_2_[Re] with ^i^Si diameters of 3.0 nm (blue) and 3.9 nm (yellow). All samples are prepared under argon with 10 μM NC in a 1:4 mixture of toluene/THF. Fits shown in bold lines and full results are shown in Figure S3 and Table S1.

We next undertake a series of TAS measurements on 3.0 and 3.9 nm ^i^Si–OCH_2_[Re], and the results are compared to those obtained for dodecyl-terminated samples lacking any surface [Re] (^i^Si–C_12_, Figures 1 and S3). All samples are prepared under argon with 10 μM NC in a 1:4 mixture of toluene/ THF. The transient spectral features are dominated by the broadly absorbing, long-lived (>5 ns) Si exciton for both ^i^Si–OCH_2_[Re] assemblies and ^i^Si–C_12_. Interestingly, on the picosecond timescale, the smaller 3.0 nm diameter samples are described by a slower component (τ_3_) relative to their 3.9 nm counterparts, where τ_3_ is 30–40% faster for ^i^Si–OCH_2_[Re] compared to ^i^Si–C_12_ for both NC sizes (Table S1). These data are consistent with an increase in direct gap character for smaller Si NCs^32^ as well as partial but very limited quenching of the NC exciton by surface-bound [Re] consistent with misaligned energy levels. For highly efficient photoinduced electron transfer from the Si NC to the [Re], we would expect the exciton quenching to be comprehensive and the steady-state PL signal to drop to 0. Instead, these TAS data suggest inefficient electron transfer from the NC to tethered [Re]. This result shows that increasing the Si NC E_CB_ to higher energy may be a marginally successful strategy to enhance NC to [Re] energy or electron transfer within the *Si–OCH_2_– attachment scheme.

**Figure 2:**
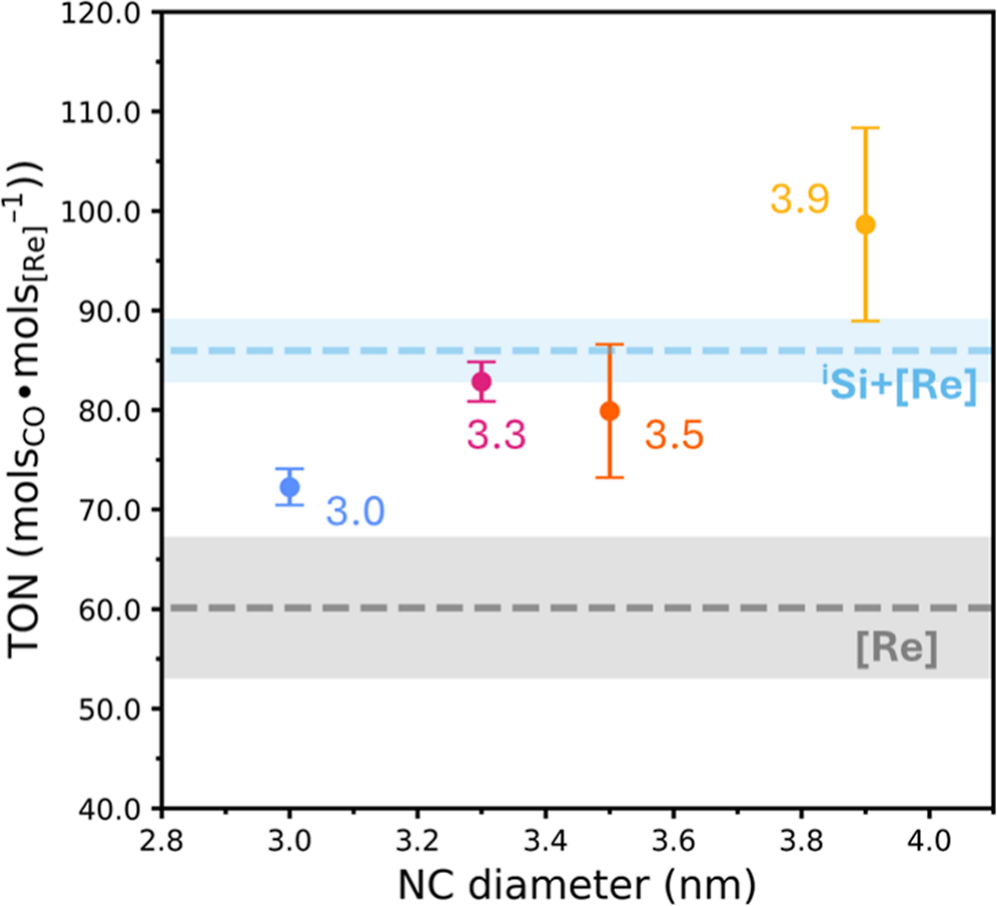
Photocatalytic CO production for ^i^Si–OCH_2_[Re] in the range of 3.0–3.9 nm at 1 μM NC compared with a mixture 1 μM in 3.9 nm ^i^Si–C_12_ and 1 μM in [Re(bpy)(CO)_3_Br] (^i^Si + [Re], blue dashed line) or [Re(bpy)(CO)_3_Br] (1 μM) with no Si NC present ([Re], black dashed line). Error for the ^i^Si + [Re] and [Re] are represented by the shaded regions. All photolyses were performed in CO_2_-saturated THF with 100 mM TFE and saturated BIH. Products in the headspace were sampled after 20 h irradiation with a 405 nm LED array.

We next explore the photocatalytic performance of ^i^Si–OCH_2_[Re]. We first quantify the [Re] loading on each assembly utilizing transmission Fourier transform infrared spectroscopy (FTIR) of 50 μM solutions of the ^i^Si–OCH_2_[Re] in toluene (Figure S4). The characteristic *fac*-Re(CO)_3_ motif, corresponding to the in-phase symmetric, equatorial asymmetric, and out-of-phase symmetric stretches of the CO ligand motif are compared to a calibration curve of [Re] in THF. From this calibration curve, we determine values of 1.6, 2.3, 2.5, and 2.4 [Re] per NC for 3.0, 3.3, 3.5, and 3.9 nm assemblies, respectively. Using these [Re] loadings, we measure the CO_2_RR photocatalytic activity of each size assembly in tetrahydrofuran (THF) under irradiation from a 405 nm LED array with 1,3-dimethyl-2-phenyl-2,3-dihydro-1*H*-benzimidazole (BIH) as a sacrificial reductant and 2,2,2-trifluoroethanol (TFE) as a proton source. In our previous report, we observed that the photocatalysis TON approached its maximum by ∼20 h for both ^i^Si–[Re] and free [Re],^27^ which is why this time was chosen for this study. Gas products are measured by gas chromatography (GC) and compared to a standard calibration curve. In all cases, CO is the major product, with CH_4_ and H_2_ also detected as minor products (Figures 2 and S5). Notably, Si–C_12_ NCs possess no inherent propensity toward catalytic CO_2_ reduction to CO;^27^ thus, we consider all CO measured as a product of [Re] catalytic turnover with or without assistance from Si. We hypothesize that the observed CH_4_ is due to formal hydride transfer from BIH based on a previous report showing that benzimidazole organic hydrides react with transition metal carbonyl complexes to generate methanol,^33^where an additional 2-electron, 2-proton reduction could generate methane. We compare the CO products formed under irradiation of ^i^Si–OCH_2_[Re] to a control sample of dissolved [Re(bpy)(CO)_3_Br] ([Re], [Re(n)L], or [Re(n)X] where n designates the Re formal oxidation state and L or X are L- or X-type ligands, respectively) or a physical mixture of untethered 3.9 nm ^i^Si–C_12_ and [Re(bpy)(CO)_3_Br] (Si + [Re]). For all assemblies and the comixture ^i^Si + [Re], there is a marked enhancement in CO_2_RR products formed over 20 h of irradiation (Figures 2 and S5) relative to [Re] only measurements. However, the 3.0 nm assembly shows a distinct drop in CO evolved compared to the larger NC assemblies. This is likely due to the blue-shifted absorption profile of 3.0 nm ^i^Si NC relative to the larger NCs (Figure S2); thus, for even smaller NC assemblies, the TON should converge to that of the [Re] control. These data confirm that NC-to-[Re] energy or electron transfer does play a minor a role in product formation. However, the process is highly inefficient, and most Si NC photoexcitation events lead to nonproductive relaxation pathways that serve to absorb light parasitically from photocatalytic [Re]. We hypothesize that there is a significant barrier to electron or energy transfer through the insulating dodecyl matrix and the saturated methylene silyl ether tether, suppressing photon-to-product formation. We conclude that a hybrid structure based on ^i^Si NCs terminated with C_12_ ligands and [Re] via short-chain tethers may restrict the appropriate symmetry for electronic coupling between the Si semiconductor and molecular species as was elegantly laid out by Rose.^34^ A visualization of the restricted degrees of freedom preventing orbital symmetry alignment within ^i^Si–[Re] is provided in Figure S6.

### Reorientation of NC Exciton by Band Edge Tuning

To further understand the size-dependent changes in the ^i^Si–OCH_2_[Re] electronic structure, we undertake a series of DFT studies. It has been reported in dark electrocatalytic systems for HER that direct electronic coupling between an electrode and bound molecular catalyst can enhance catalytic activity^35−37^ that has been ascribed to a concerted proton-coupled electron transfer (PCET) process.^38^ Thus, our aim is to better understand the inherent energetic relationship between the Si NC and surface [Re] complexes and to identify systems possessing strong coupling between the silicon band structure and [Re]-centered orbitals. Si NCs in the range of our synthetically accessible 3.0–3.9 nm diameter series comprise between 700 and 1600 Si atoms, too computationally expensive for the standard DFT methods used herein. Instead, small cluster models are utilized for hydride-terminated Si NCs (^i^Si–H) or methyl-terminated Si NCs (^i^Si–Me) between 1.4 and 2.0 nm diameter consisting of 71–191 Si atoms, respectively. The models are generated in python by carving a sphere of the desired diameter from a block of diamond silicon. Surface hydrides are added with Avogadro, and all DFT is run using the Orca 5.0.3 suite at the M06-L/TZVP/SVP level of theory. Quantum confinement models for nanocrystal band gap energy follow a power law relationship and have been used by our group and others to track size-dependent changes in the semiconductor band structure according to eq 1
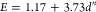
1where *E* is energy in eV and *d* is the NC diameter in nm. Estimates for quantum-confined Si according to the effective mass approximation (EMA) suggested a power law trend according to *d*^–2^ for silicon.^28,39,40^ Alternately, Delerue et al. proposed variation from the EMA predictions at smaller nanocrystal sizes according to a *d*^–1.39^ trend using a linear combination of atomic orbitals (LCAO) approach.^40^ In our previous work, a set of experimentally derived ^i^Si NC band gap energies was fit to eq 2 and found a *d*^–1.69^ trend for dodecyl-terminated ^i^Si NCs (black dashed line, Figure S7) intermediate to the predicted EMA/LCAO bounds (yellow-shaded region, Figure S7).^28^
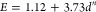
2

Our computational results are checked by plotting Δ*E* = *E*_CB_ – *E*_VB_ vs model diameter for our series of 1.4–2.0 nm clusters and fitting it to the power law relationship (eq 2). A *d*^–1.38^ trend for these ultrasmall ^i^Si–H clusters in good agreement with the LCAO approach (blue solid line, Figure S7).

**Figure 3:**
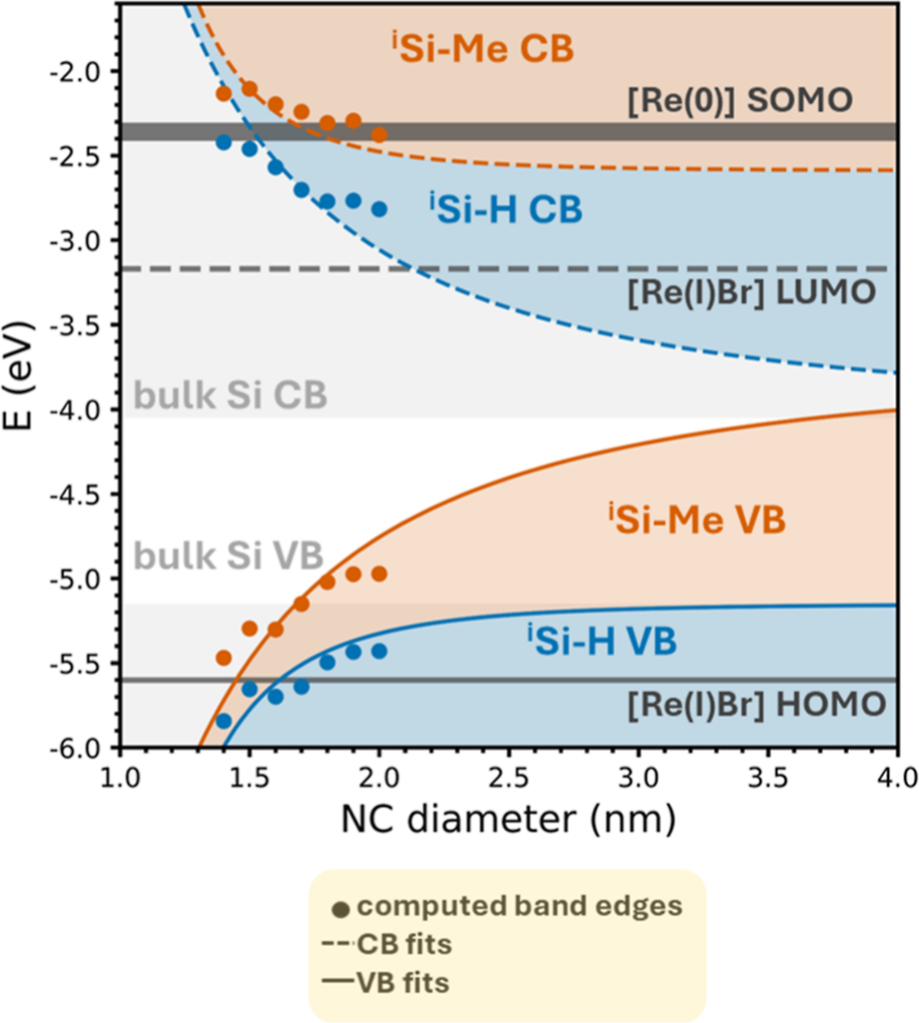
DFT-predicted band edges (circles) as a function of NC model size compared to tethered [Re] FMOs (dark gray) and bulk silicon band edges (light gray). The red (^i^Si–C_12_) and blue (^i^Si–H) shaded regions correspond to NC diameter-dependent fits according to the DFT predicted band edges according to eq 2.

We next compare the absolute band edge positions for ^i^Si–H and ^i^Si–Me to bulk silicon and independently calculate [Re] frontier molecular orbitals (FMOs). We fit the DFT-predicted CB and VB band edges for both models to a similar power law as above, constraining the function to converge at experimentally determined bulk band edge energies^22^ according to eqs 3 and 4
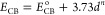
3
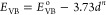
4where *E*_CB_ and *E*_VB_ are the predicted conduction and valence band edge energies (respectively, in eV), and *E*_CB_^o^ and *E*_VB_^o^ are the bulk band edge energies (in eV). As shown in Figure 3 (blue points and fits), the ^i^Si–H band edges are constrained to −4.05 eV (*E*_CB_) and −5.15 eV (*E*_VB_) to model progression toward the bulk silicon bands at larger NC sizes (*d* > ∼10 nm).^41^

However, the influence of alkyl termination on the ^i^Si–Me band edges requires a different treatment due to the electronic influence of surface alkyl groups on the electron affinity and ionization potential relative to vacuum. In this case, *E*_VB_ for ^i^Si–Me is constrained to −3.71 eV and *E*_CB_ to −2.59 eV based on these experimentally determined values for hexyl-terminated bulk silicon from Huffman et al.^22^ These constraints give an excellent match to our computed band edges; when we plot the difference between the CB (orange dashed line, Figure 3) and VB (orange solid line, Figure 3) fits for ^i^Si–Me clusters against NC diameter from 1 to 10 nm (maroon trace, Figure S7), they follow a *d*^–1.58^ relationship according to eq 2, also in close agreement with our experimentally determined trend (black dashed trace, Figure S7).

Comparing the ^i^Si–Me *E*_CB_ energy (orange fit) to the [Re(I)Br] LUMO energy (dark gray dashed line, Figure 3) suggests that the ^i^Si–Me *E*_CB_ resides well above the [Re(I)Br] LUMO for all NC diameters. Therefore, electron transfer from the NC to [Re(I)Br] is feasible for the first electron transfer event in ^i^Si–OCH_2_[Re] hybrids. However, we also calculate the energy level of the resulting one-electron reduced [Re(0)] with either a native Br^–^ ligand ([Re(0)Br]^−^) or CH_3_CN solvento ligand ([Re(0)CH_3_CN]^0^) and find that the empty electronic state of the singly occupied molecular orbital (SOMO, also referred to as the β-LUMO) for both the bromide and solvento complexes resides above *E*_CB_ for ^i^Si–Me for NC larger than ∼2.0 nm (dark gray bold line, Figure 3). The two electron reduced [Re(−1)] complex is generally accepted as the key intermediate in catalytic CO_2_RR based on its rapid reactivity with CO_2_ tracked by stop-flow kinetic measurements.^42^ We thus consider that photoinduced electron transfer in ^i^Si–OCH_2_[Re] assemblies is limited to formation of the one electron reduced [Re(0)] complex instead of the CO_2_RR-active [Re(−1)] state. These DFT data provide a sound rationale for why TAS shows no benefit of the hybrid structure.

### Band–Orbital Mixing in Si–[Re] Hybrids

Previously, our group reported band edge tuning of Si NCs by hybridization with different ligands,^29,43^ incorporation of electronic dopants,^44,45^ or both.^46^ With these two levers in mind, we undertake a series of DFT studies for assemblies of ^i^Si or B:Si NCs with [Re] complexes bound by various tethering chemistries (Scheme S1). Since varying the attachment chemistry is unlikely to move the [Re(0)] SOMO to a sufficiently low energy level below the ^i^Si NC conduction band (the core bpy and ancillary ligands largely control the [Re] states), we focus here on comparing nonconjugated versus conjugated linkages in generating sufficiently strong coupling between the Si and [Re] to form new hybridized states. This strategy has precedent in coupling arenes to the electronic structure of Si NCs as has been shown to enable upconversion^47^ and band gap modulation in Si NC-arene hybrids.^48,49^ In addition to the methylene silyl ether anchor, we also consider an ethylene bridge (*Si–C_2_H_4_[Re]), a direct Si–bpy bond (*Si–C_Ar_[Re]), or a bipyrazine complex (*Si–N_Ar_[Re]) with distal N donors as a Lewis base for binding to surface B sites for the B:Si system only.

**Figure 4:**
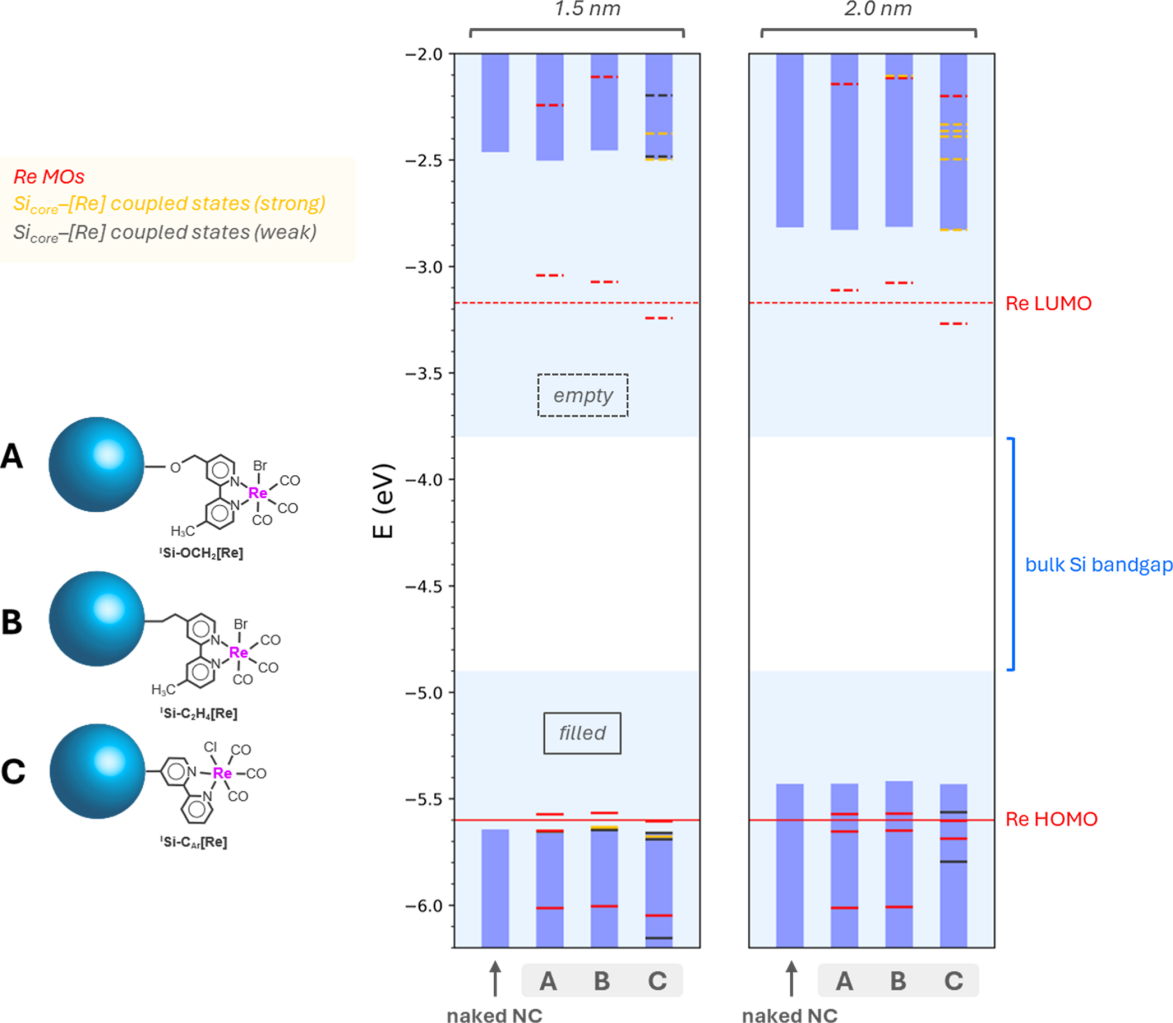
DFT-predicted energetics of 1.5 and 2.0 nm ^i^Si–OCH_2_[Re] models with the shown surface attachment chemistries (A–C). Blue-shaded bars represent the B:Si NC bands, and the light blue-shaded regions represent the bulk Si band structure. Strongly coupled Si–[Re] states are shown as gold lines, [Re]-centered orbitals are shown as red lines, and slightly coupled states are shown as black or salmon lines for Si- or [Re]-dominant orbitals, respectively. Dashed lines represent empty orbitals, and solid lines represent filled orbitals, in the ground state. The unbound [Re(bpy)(CO)_3_Br] HOMO and LUMO are shown as solid and dashed red lines, respectively, across the entire plot.

As expected, the models with ^i^Si NCs do not predict any coupling for either of the saturated linker systems and only show strong electronic interaction in the ^i^Si–C_Ar_[Re] model (type C, yellow dashed lines, Figure 4). Therefore, we identify ^i^Si–C_Ar_[Re] as a good target moving forward. However, achieving the *Si–C_Ar_ linkage is a significant synthetic challenge using conventional thermal radical chemistry since the precursor salt, [Re(N_2_bpy)(CO)_3_Cl]BF_4_ where N_2_bpy = 2,2’- bipyridyl-4-diazonium ([Re]N_2_),^50^ is thermally unstable and insoluble in toluene. Thus, we found this [Re]N_2_ precursor complex is incompatible with the tethering chemistry employed for the preparation of ^i^Si–OCH_2_[Re] samples. Instead, we target B:Si–C_Ar_[Re] to compare to our previous B:Si–OCH_2_[Re] results described above that is amenable to nonthermal surface functionalization.

**Figure 5:**
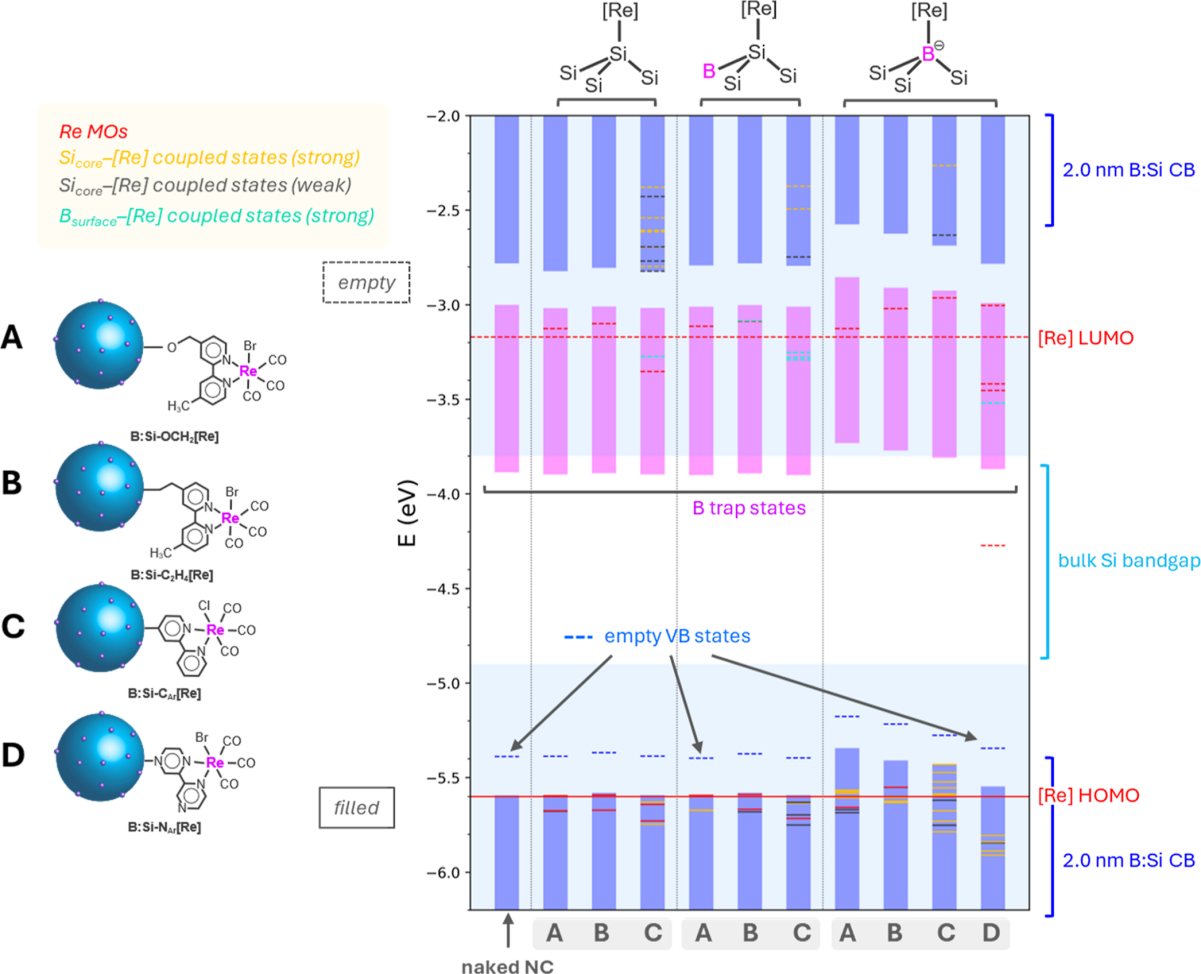
DFT-predicted energetics of 2.0 nm B:Si–[Re] models with various considered tethers (A–D). Blue-shaded bars represent the B:Si NC bands, pink-shaded bars represent B intra-band-gap trap states, and the light blue-shaded regions represent the bulk Si band structure. Strongly coupled Si–[Re] states are shown as gold lines, [Re] centered orbitals are shown as red lines, slightly coupled states are shown as black or salmon lines for Si- or [Re]-dominant orbitals, respectively, and coupled B–[Re] states are shown as pink lines. Dashed lines represent empty orbitals and solid lines represent filled orbitals. The unbound [Re(bpy)(CO)_3_Br] HOMO and LUMO are shown as a solid or dashed red line, respectively, across the entire plot.

To fully account for the more complex surface chemistry in B:Si NCs, we consider [Re] complexes bound to three different surface sites: (1) *Si bonded to three other Si atoms ({Si_3_}*Si), (2) *Si bonded to two Si atoms and a single B atom ({BSi_2_}*Si), or (3) a surface Lewis acidic B* site ({Si_3_}*B). We again consider both 1.5 (Figure S9) and 2.0 (Figure 5) nm diameter model systems and find consistent trends for both sizes. From our computational results, it is clear that, similar to the intrinsic system, B:Si–C_Ar_[Re] achieves the greatest coupling of any of the linkage chemistries. We predict no coupling in the CB states at any anchor site for the saturated linkers, B:Si–OCH_2_[Re] and B:Si–C_2_H_4_[Re]. B:Si–N_Ar_[Re] also does not exhibit coupling in the CB states. Interestingly, the bpz complex LUMO (−4.27 eV for the 2.0 nm model) is situated well below that of other Si–[Re] hybrids and below the CB of bulk silicon (−3.8 eV),^51^ suggesting that downhill electron transfer should occur from excited-state Si to [Re] in the B:Si–N_Ar_[Re] systems. However, in the context of CO_2_ reducing assemblies, the low-level LUMO predicted here lies well below the CO_2_/CO reduction potential (ca. −4 eV at pH 7.0). In general, it appears that regardless of the tethering group, anchoring to {Si_3_}*B sites induces coupling only in the VB, and thus, tethers relying solely on L-type ligation are unsuitable for coupled systems. The results from the DFT modeling are clear that the *Si–C_Ar_[Re] linkage represents the best target for a coupled system in both ^i^Si and B:Si systems.

### Electronic Investigation of Theoretical Targets

To validate the DFT predictions, we synthesize two different B:Si–[Re] hybrid systems. As previously reported by our group,^44,46^ B:Si NCs spontaneously dissolve into polar organic solvents such as dimethyl sulfoxide (DMSO) due to Lewis acid–base interactions as well as covalent attachment. Here, we find similar dissolution is possible using CH_3_CN solvent with sonication to effectively dissolve naked B:Si NCs that we term B:Si–CH_3_CN and use as a catalyst-free control sample. Sonication has also been shown as an effective method for binding ligand precursors to a bulk silicon surface,^22^ and we surmise that sonication also may achieve covalent tethering chemistry of [Re] complexes to B:Si NCs. To generate B:Si–OCH_2_[Re] and B:Si–C_Ar_[Re], a solution of [Re]CHO or [Re]N_2_ is sonicated in CH_3_CN with a stoichiometric amount of 3.9 nm B:Si NC powder for a total of 2 h. In both cases, the product solution is a dark coffee-colored colloid. The solution is filtered through a 0.7 μm plug to remove any insoluble B:Si NCs. These samples are incompatible with the standard solvent/antisolvent purification since the solids do not redisperse after precipitation. Dried films of both samples are characterized by DRIFTS (Figure S10), and each shows a large signal at ∼2080 cm^–1^ corresponding to the Si–H stretch, with a shoulder at ∼2020 cm^–1^ and a peak at 1930 cm^–1^ (blue dashed trace, B:Si–OCH_2_[Re]) or 1954 cm^–1^ (red trace, B:Si–C_Ar_[Re]), indicating the presence of both B:Si and the *fac*-Re(CO)_3_ motif in the films. The DRIFTS spectrum of B:Si–OCH_2_[Re] additionally shows loss of the [Re]CHO aldehyde peak, suggesting successful insertion of the ligand-centered aldehyde into surface hydrides. The B:Si–C_Ar_[Re] film is also compared to a DRIFTS spectrum of sonically degraded [Re]N_2_ and an ATR spectrum of pristine [Re]N_2_ powder (Figure S11). The low-energy *fac*-Re(CO)_3_ peak of B:Si–C_Ar_[Re] is blue-shifted from either the pristine or sonically degraded controls of [Re]N_2_, confirming successful anchoring.

**Figure 6:**
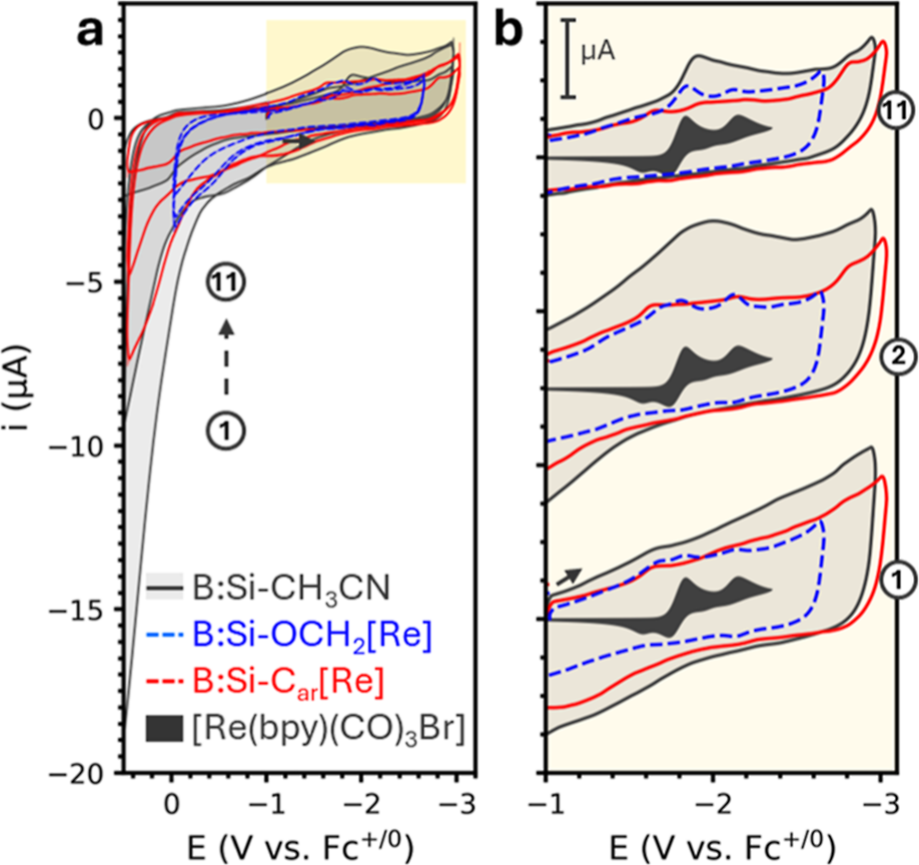
(a) CVs of B:Si–CH_3_CN (black line, gray shading), B:Si–OCH_2_[Re] (blue dashed trace), and B:Si–C_Ar_[Re] (red solid line) thin films on glassy carbon. The 1st, 2nd, and 11th of 11 repeat cycles are shown. (b) The same voltammograms zoomed in on the cathodic region and vertically offset for clarity. A voltammogram of dissolved [Re(bpy)(CO)_3_Br] (black-shaded trace) is included for comparison. All voltammograms are collected at 50 mV s^–1^ in CH_3_CN under an argon atmosphere. The [Re(bpy)(CO)_3_Br] voltammogram was collected with a freshly polished glassy carbon electrode in the presence of 1 mM [Re(bpy)(CO)_3_Br] and scaled to 1/12th for clarity.

To understand the electrochemical behavior of B:Si–CH_3_CN, B:Si–OCH_2_[Re], and B:Si–C_Ar_[Re], we subject thin films of each to cyclic voltammetry (CV, Figures 6, S12, and S13). We compare these data to a sample comprising a physical mixture (1:1) of B:Si–CH_3_CN and degraded [Re]N_2_ by sonicating B:Si NC powder in a 100 μM solution of freshly degraded [Re]N_2_ and refer to this sample as B:Si + [Re]N_2_. A detailed CV analysis is included in the Supporting Information. Ultimately, we observe features of free [Re(bpy)(CO)_3_Br] in B:Si–OCH_2_[Re] samples (blue traces in Figures 6, S12, and S13) and B:Si + [Re]N_2_ (yellow trace, Figure S13) but no relevant peaks in B:Si–C_Ar_[Re] (red traces in Figures 6, S12, and S13). From these results, we conclude that both B:Si–OCH_2_[Re] and B:Si + [Re]N_2_ show the uncoupled reduction of both B:Si (*E*_p,c_ ca. −1.66 V vs Fc^+/0^) and [Re] (*E*_p,c1_ ca. −1.8, −2.15 V vs Fc^+/0^), whereas B:Si–C_Ar_[Re] exhibits no signs of [Re] reduction that suggests coupling between [Re] and Si states.

**Figure 7:**
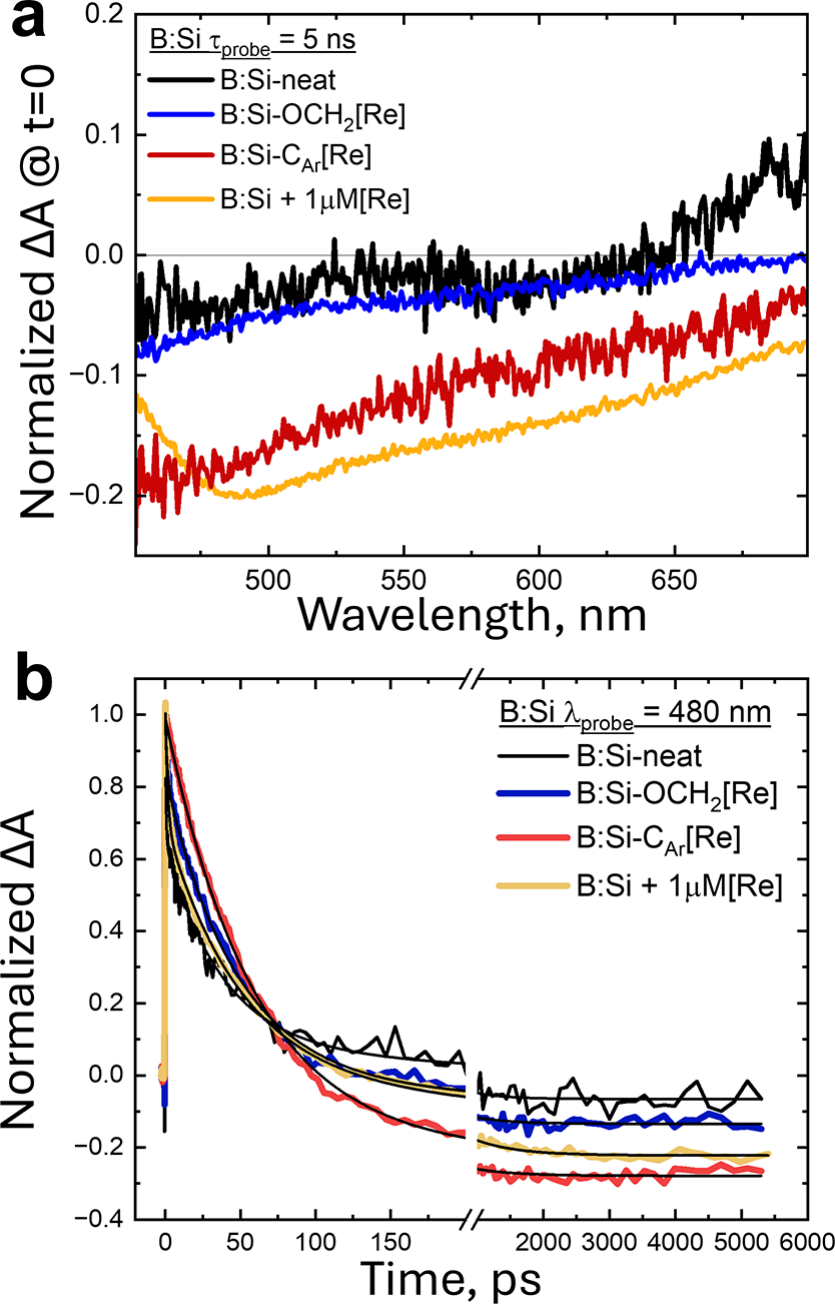
Transient absorption data generated by a 400 nm pump pulse. (a) Transient absorption spectra of B:Si–CH_3_CN (black), B:Si–OCH_2_[Re] (blue), a physical mixture of B:Si–CH_3_CN and [Re(bpy)(CO_3_)Br] (yellow), and B:Si–C_Ar_[Re] (red) at a pump–probe delay of 5 ns. (b) Transient dynamics of B:Si–CH_3_CN (black), B:Si–OCH_2_[Re] (blue), a physical mixture of B:Si–CH_3_CN and [Re(bpy)(CO_3_)Br] (yellow), and B:Si–C_Ar_[Re] (red) at λ_probe_ = 480 nm. Time-dependent spectra for all samples shown here can be found in Figure S14a–d. All samples collected with 1 μM analyte(s) in Ar-saturated CH_3_CN.

We next probe the transient behavior of colloidal B:Si–CH_3_CN, B:Si–OCH_2_[Re], and B:Si–C_Ar_[Re] upon 405 nm excitation in CH_3_CN (Figures 7 and S13). Excitation of B:Si–CH_3_CN induces a broad absorption within the first 290 fs, which relaxes within 5 ns. This recombination is much faster than that observed for ^i^Si–C_12_ samples and is consistent with the lack of photoluminescence for B:Si NC. We next compare the B:Si–CH_3_CN transient dynamics to solutions composed of B:Si–CH_3_CN (1 μM) and untethered [Re(bpy)(CO)_3_Br] (1 μM or 100 μM) in CH_3_CN (Figures 7 and S14). The time-resolved dynamics of [Re(bpy)(CO)_3_Br] are well-known in the literature,^52^ which are important to review briefly since the absence of the long-lived exciton observed for ^i^Si–OCH_2_[Re] means that the photodynamics in Figure 7 are a convolution of those from [Re(bpy)(CO)_3_Br] as well as hybrid B:Si–OCH_2_[Re] and spatially associated B:Si ↔ [Re] complexes.

Briefly, excitation of the MLCT band (λ_max_ = 375 nm) results in rapid intersystem crossing from the Re-centered singlet state to the space-separated triplet state with tens of ns lifetime at ambient temperature. The triplet signal is characterized by a sharp photoinduced absorption at λ_max_ ∼ 480 nm with a broad peak at λ_max_ ∼ 570 nm that exhibits little to no decay on the 0.3–5000 ps timescale of our experiment. The [Re(bpy)(CO)_3_Br] spectral signature (Figure S14e) dominates the solution of B:Si–CH_3_CN with a large excess of [Re(bpy)(CO)_3_Br] (100 mM, Figure S14f). However, 1:1 solutions of B:Si–CH_3_CN (1 μM) and [Re(bpy)(CO)_3_Br] (1 μM) reveal a new interaction between B:Si–CH_3_CN and [Re(bpy)(CO)_3_Br] (Figure S14d). A bleach of the transient [Re(bpy)(CO)_3_Br] signal is revealed between 50 and 500 ps after complete relaxation of the B:Si–CH_3_CN exciton due to the presence of [Re(bpy)(CO)_3_Br] in solution. Ultrafast charge transfer from NC systems to untethered metal complexes is a well-documented phenomenon in the literature.^53−55^ The sub-nanosecond excited-state electron transfer event is facilitated by preassociation between the NC and the metal complex. We posit that the long-lived bleach signal is the result of a subpopulation of the [Re] ↔ B:Si NC association complex, only revealed after complete relaxation of unassociated B:Si–CH_3_CN. It is likely that the preassociation complex forms by a flat interaction where the NC bands can mix with the bpy π system, a phenomenon well recorded in the literature.^56^

The [Re] tethered via the saturated linker in B:Si–OCH_2_[Re] imparts minimal effect on the temporal evolution of the B:Si NC photoinduced absorption cf. neat B:Si. Complete relaxation of the transient signal is achieved within 500 ps with minimal bleach observed. Thus, we conclude that limited association exists between tethered –OCH_2_[Re] and the B:Si NC, likely due to spatial restrictions imposed by the two-atom linker that prevents the required flat interaction by the bpy π system. This agrees with the observed behavior from ^i^Si–OCH_2_[Re] (Figure 1) showing that the –OCH_2_– linker severely hinders electronic communication between Si and [Re].

**Figure 8:**
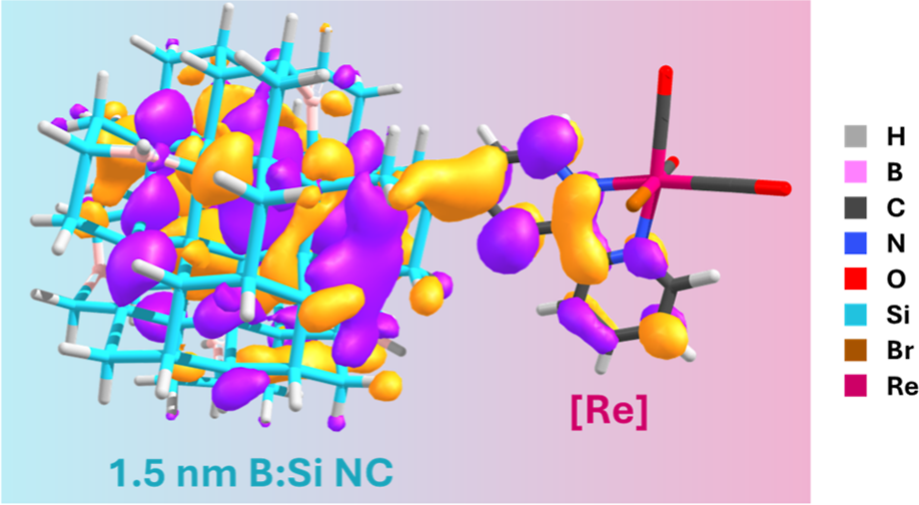
Computationally predicted orbital density plot for the empty 736α (Si LUMO) orbital of 1.5 nm B:Si–C_Ar_[Re].

The magnitude of this bleach increases for B:Si–C_Ar_[Re], suggesting that there is a stronger interaction between B:Si and [Re] with this linker. The transient decay dynamics of B:Si–C_Ar_[Re] evolve over time in a manner clearly distinct from that of ^i^Si–C_12_ and ^i^Si–OCH_2_[Re], as shown in Figure 1. The initial time point scan reveals a broad photoinduced absorption throughout the visible window. This feature decays at the same rate as the B:Si–OCH_2_[Re] signal monitored at the same wavelength over the course of the experiment, and thus, we assign it to the B:Si exciton. Monitored at 480 nm, there is an early time point growth in the signal over the first ps followed by decay into a bleached [Re]-centered signal between 50 and 500 ps. The homology between the untethered mixture of B:Si and [Re] and the transient decay kinetics of B:Si–C_Ar_[Re] suggests that both cases involve band-FMO mixing between the NC and [Re]. While the specific tethering moiety of B:Si–C_Ar_[Re] is less flexible than B:Si–OCH_2_[Re], direct orbital interaction through the tethered bpy π system is feasible, as predicted computationally above (Figure 5) and observed visually in a representative orbital density plot for a strongly coupled Si_core_–[Re] state (Figure 8). This coupled interaction should provide a convenient pathway for photoinduced electron transfer from the NC to the bound [Re] complex. However, the TAS data show that this coupling between the [Re] orbital structure and the NC bands additionally opens a new pathway for excitonic relaxation that is undesirable for photocatalysis.

### Photocatalysis of B:Si–[Re] Hybrids

Finally, we compare the catalytic activity of colloidal B:Si–C_Ar_[Re] (1 μM) to that recorded for B:Si–OCH_2_[Re] (1:1, 1 μM) and free [Re(bpy)(CO)_3_Br] (1 μM) in CH_3_CN (vide supra, Figure S15). We note that the conditions employed for photocatalysis in the B:Si NC systems are different from those discussed earlier for the ^i^Si systems (in particular, CH_3_CN vs THF solvent), making direct comparison difficult. Instead, we focus on relative changes between samples measured under these conditions. The catalytic measurements probe the practical effects of coupling on CO_2_RR and which of the two competing forces—photoinduced energy and/or electron transfer leading to productive photocatalysis versus enhanced recombination—win out in the electronically coupled B:Si–C_Ar_[Re] hybrid. After 20 h of photolysis by a 405 nm LED array, CO, H_2_, and CH_4_ are detected in the headspace. However, the TON of CO produced by B:Si–C_Ar_[Re] is 2–3 times lower than that of either B:Si–OCH_2_[Re] or [Re(bpy)(CO)_3_Br] under the same conditions. Since B:Si–C_Ar_[Re] features an axial chloride ligand instead of bromide ligand, comparison to the control bromide complex used for catalytic measurements should provide an overestimate of the assembly’s catalytic activity since chloride complexes are known to exhibit faster turnover than the corresponding bromide complexes.^57^ Instead, worse TON for CO_2_RR to CO is found. Since results from our computational studies and saturated linker system suggest empty [Re] orbitals do not occupy intra-band-gap space, the photocatalytic results suggest that the B:Si–C_Ar_[Re] system is parasitically leaching photoinduced charges in the surface [Re] complex—which otherwise would be available for catalysis—into the B:Si NC through the coupled electronic states.

## Conclusions

We explored quantum confinement and doping effects on Si NC–[Re] hybrid assemblies’ energetics and catalytic activity. Following conclusions from our previous result, most observed catalytic activity proceeds via direct light absorption by surface [Re] with minimal assistance from the NC. Raising the Si conduction band energy level to increase the driving force for photoinduced electron transfer by varying the Si NC size between 3.9 and 3.0 nm is a successful strategy for ultrasmall Si NCs, but this does not affect photocatalysis in a statistically significant manner since increasing the band gap energy blue-shifts the Si absorption such that highly quantum-confined Si NCs only weakly absorb visible light.

Doping, on the other hand, provides a viable pathway to achieve the desired Si–[Re] energetic matching when the correct surface chemistry is applied. We computationally analyzed a number of B:Si–[Re] models and compared the electrochemical, spectral, and catalytic behavior of two synthetically accessible target hybrid structures. We observed evidence of coupling in the directly tethered B:Si–C_Ar_[Re] samples as predicted computationally. Though this purely photocatalytic system suffers from static driving forces depending on fixed band/orbital energies, this coupled system is intriguing for future photoelectrochemical (PEC) applications. A PEC system may allow for dynamic control of the energetics of the catalyst FMOs, and the band bending present in a bulk PEC system may mitigate recombination that appears to limit photocatalytic performance in B:Si–C_Ar_[Re]. Since many CO_2_RR molecular electrocatalysts feature high lying FMOs, development of lower redox potential CO_2_RR catalysts or the choice of a less energetically costly catalytic reaction (e.g., HER) may be required to produce a favorable match between the Si CB level and tethered catalyst vacant orbitals. Other photocathode modifications, such as oxide or other interfacial modifiers as well as using semiconductors with higher energy conduction band positions (i.e., not Si that is the focus of this work), also may be viable strategies. Ongoing work in our laboratory is exploring these options, with the goal of replicating the Si–metal complex coupling observed herein by facilitating a low barrier photocatalytic cascade from the Si NC to the surface metal complex and subsequently providing barriers to deleterious recombination.

## Supporting Information Available

Materials and methods, supporting spectra, and computational results (PDF)

## Abbreviations

NC: nanocrystal
^i^Si: intrinsic silicon
B:Si: boron-doped silicon
[Re]: Re electrocatalyst
E_CB_: conduction band energy
CO_2_RR: CO_2_ reduction reaction
HER: hydrogen evolution reaction
E_VB_: valence band energy
LCAO: linear combination of atomic orbitals
EMA: effective mass approximation
FMO: frontier molecular orbital
LUMO: lowest occupied molecular orbital
HOMO: highest occupied molecular orbital
SOMO: singularly occupied molecular orbital
DFT: density functional theory
CV: cyclic voltammetry
TAS: transient absorption spectroscopy
PECVD: plasma enhanced chemical vapor deposition
DRIFTS: diffuse reflectance infrared Fourier transform spectroscopy
FTIR: Fourier transform infrared spectroscopy
ATR: attenuated total reflectance
PL: photoluminescence
GC: gas chromatography
bpy: 2,2′-bipyridyl
mcabpy: 4-methyl-2,2′-bipyridyl-4′-carboxaldehyde
BIH: 1,3-dimethyl-2-phenyl-2,3-dihydro-1H-benzimidazole
THF: tetrahydrofuran
TFE: 2,2,2-trifluoroethanol.

## References

[ref1] Shaner M. R.; Atwater H. A.; Lewis N. S.; McFarland E. W. A comparative technoeconomic analysis of renewable hydrogen production using solar energy. Energy Environ. Sci. 2016, 9, 2354–2371. 10.1039/C5EE02573G.

[ref2] Spitler M. T.; Modestino M. A.; Deutsch T. G.; Xiang C. X.; Durrant J. R.; Esposito D. V.; Haussener S.; Maldonado S.; Sharp I. D.; Parkinson B. A.; Ginley D. S.; Houle F. A.; Hannappel T.; Neale N. R.; Nocera D. G.; McIntyre P. C. Practical challenges in the development of photoelectrochemical solar fuels production. Sustain. Energy Fuels 2020, 4, 985–995. 10.1039/C9SE00869A.

[ref3] Wang Q.; Pornrungroj C.; Linley S.; Reisner E. Strategies to improve light utilization in solar fuel synthesis. Nat. Energy 2022, 7, 13–24. 10.1038/s41560-021-00919-1.

[ref4] Ballif C.; Haug F.-J.; Boccard M.; Verlinden P. J.; Hahn G. Status and perspectives of crystalline silicon photovoltaics in research and industry. Nat. Rev. Mater. 2022, 7, 597–616. 10.1038/s41578-022-00423-2.

[ref5] Bookbinder D. C.; Lewis N. S.; Bradley M. G.; Bocarsly A. B.; Wrighton M. S. Photoelectrochemical Reduction of N,N’-Dimethyl-4,4’-bipyridinium in Aqueous Mediaat p-Type Silicon: Sustained Photogeneration of a Species Capable of Evolving Hydrogen. J. Am. Chem. Soc. 1979, 101, 7721–7723. 10.1021/ja00520a019.

[ref6] Walter M. G.; Warren E. L.; McKone J. R.; Boettcher S. W.; Mi Q.; Santori E. A.; Lewis N. S. Solar Water Splitting Cells. Chem. Rev. 2010, 110, 6446–6473. 10.1021/cr1002326. 21062097

[ref7] Lichterman M. F.; Hu S.; Richter M. H.; Crumlin E. J.; Axnanda S.; Favaro M.; Drisdell W.; Hussain Z.; Mayer T.; Brunschwig B. S.; Lewis N. S.; Liu Z.; Lewerenz H.-J. Direct observation of the energetics at a semiconductor/liquid junction by operando X-ray photoelectron spectroscopy. Energy Environ. Sci. 2015, 8, 2409–2416. 10.1039/C5EE01014D.

[ref8] Peng F.; Wang J.; Ge G.; He T.; Cao L.; He Y.; Ma H.; Sun S. Photochemical reduction of CO2 catalyzed by silicon nanocrystals produced by high energy ball milling. Mater. Lett. 2013, 92, 65–67. 10.1016/j.matlet.2012.10.059.

[ref9] Dasog M.; Kraus S.; Sinelnikov R.; Veinot J. G.; Rieger B. CO2 to methanol conversion using hydride terminated porous silicon nanoparticles. Chem. Commun. 2017, 53, 3114–3117. 10.1039/C7CC00125H. 28245018

[ref10] Sun W.; Qian C.; He L.; Ghuman K. K.; Wong A. P.; Jia J.; Ali F. M.; O’Brien P. G.; Reyes L. M.; Wood T. E.; Helmy A. S.; Mims C. A.; Singh C. V.; Ozin G. A. Heterogeneous reduction of carbon dioxide by hydride-terminated silicon nanocrystals. Nat. Commun. 2016, 7, 12553 10.1038/ncomms12553. 27550234 PMC4996982

[ref11] Neiner D.; Kauzlarich S. M. Hydrogen-Capped Silicon Nanoparticles as a Potential Hydrogen Storage Material: Synthesis, Characterization, and Hydrogen Release. Chem. Mater. 2010, 22, 487–493. 10.1021/cm903054s.

[ref12] Bookbinder D. C.; Bruce J. A.; Dominey R. N.; Lewis N. S.; Wrighton M. S. Synthesis and characterization of a photosensitive interface for hydrogen generation: Chemically modified p-type semiconducting silicon photocathodes. Proc. Natl. Acad. Sci. U.S.A. 1980, 77, 6280–6284. 10.1073/pnas.77.11.6280. 16592907 PMC350266

[ref13] Bocarsly A. B.; Bookbinder D. C.; Dominey R. N.; Lewis N. S.; Wrighton M. S. Photoreduction at Illuminated p-Type Semiconducting Silicon Photoelectrodes. Evidence for Fermi Level Pinning. J. Am. Chem. Soc. 1980, 102, 3683–3688. 10.1021/ja00531a003.

[ref14] Seo J.; Pekarek R. T.; Rose M. J. Photoelectrochemical operation of a surface-bound, nickel-phosphine H_2_ evolution catalyst on p-Si(111): a molecular semiconductor|catalyst construct. Chem. Commun. 2015, 51, 13264–13267. 10.1039/C5CC02802G. 25999134

[ref15] Kim H. J.; Seo J.; Rose M. J. H_2_ Photogeneration Using a Phosphonate-Anchored Ni-PNP Catalyst on a Band-Edge-Modified p-Si(111)|AZO Construct. ACS Appl. Mater. Interfaces 2016, 8, 1061–1066. 10.1021/acsami.5b09902. 26741653

[ref16] Dempsey J. L.; Heyer C. M.; Meyer G. J. A Vision for Sustainable Energy: The Center for Hybrid Approaches in Solar Energy to Liquid Fuels (CHASE). Electrochem. Soc. Interface 2021, 30, 65–68. 10.1149/2.F10211IF.

[ref17] Kaufman A. J.; Nielander A. C.; Meyer G. J.; Maldonado S.; Ardo S.; Boettcher S. W. Absolute band-edge energies are over-emphasized in the design of photoelectrochemical materials. Nat. Catal. 2024, 7, 615–623. 10.1038/s41929-024-01161-0.

[ref18] Wang Q.; Warnan J.; Rodríguez-Jiménez S.; Leung J. J.; Kalathil S.; Andrei V.; Domen K.; Reisner E. Molecularly engineered photocatalyst sheet for scalable solar formate production from carbon dioxide and water. Nat. Energy 2020, 5, 703–710. 10.1038/s41560-020-0678-6.

[ref19] Rahaman M.; Pulignani C.; Miller M.; Bhattacharjee S.; Bin Mohamad Annuar A.; Manuel R. R.; Pereira I. A. C.; Reisner E. Solar-Driven Paired CO2 Reduction–Alcohol Oxidation Using Semiartificial Suspension, Photocatalyst Sheet, and Photoelectrochemical Devices. J. Am. Chem. Soc. 2025, 147, 8168–8177. 10.1021/jacs.4c10519. 40020032 PMC11912307

[ref20] Shang B.; Zhao F.; Choi C.; Jia X.; Pauly M.; Wu Y.; Tao Z.; Zhong Y.; Harmon N.; Maggard P. A.; Lian T.; Hazari N.; Wang H. Monolayer Molecular Functionalization Enabled by Acid–Base Interaction for High-Performance Photochemical CO2 Reduction. ACS Energy Lett. 2022, 7, 2265–2272. 10.1021/acsenergylett.2c01147.

[ref21] Jia X.; Cui K.; Alvarez-Hernandez J. L.; Donley C. L.; Gang A.; Hammes-Schiffer S.; Hazari N.; Jeon S.; Mayer J. M.; Nedzbala H. S.; Shang B.; Stach E. A.; Stewart-Jones E.; Wang H.; Williams A. Synthesis and Surface Attachment of Molecular Re(I) Hydride Species with Silatrane Functionalized Bipyridyl Ligands. Organometallics 2023, 42, 2238–2250. 10.1021/acs.organomet.3c00235. PMC13441430 42559585

[ref22] Huffman B. L.; Bein G. P.; Atallah H.; Donley C. L.; Alameh R. T.; Wheeler J. P.; Durand N.; Harvey A. K.; Kessinger M. C.; Chen C. Y.; Fakhraai Z.; Atkin J. M.; Castellano F. N.; Dempsey J. L. Surface Immobilization of a Re(I) Tricarbonyl Phenanthroline Complex to Si(111) through Sonochemical Hydrosilylation. ACS Appl. Mater. Interfaces 2023, 15, 984–996. 10.1021/acsami.2c17078. 36548441

[ref23] Shang B.; Rooney C. L.; Gallagher D. J.; Wang B. T.; Krayev A.; Shema H.; Leitner O.; Harmon N. J.; Xiao L.; Sheehan C.; Bottum S. R.; Gross E.; Cahoon J. F.; Mallouk T. E.; Wang H. Aqueous Photoelectrochemical CO2 Reduction to CO and Methanol over a Silicon Photocathode Functionalized with a Cobalt Phthalocyanine Molecular Catalyst. Angew. Chem., Int. Ed. 2023, 62, e202215213 10.1002/anie.202215213. 36445830

[ref24] McGuigan S.; Tereniak S. J.; Donley C. L.; Smith A.; Jeon S.; Zhao F.; Sampaio R. N.; Pauly M.; Keller L.; Collins L.; Parsons G. N.; Lian T.; Stach E. A.; Maggard P. A. Discovery of a Hybrid System for Photocatalytic CO2 Reduction via Attachment of a Molecular Cobalt-Quaterpyridine Complex to a Crystalline Carbon Nitride. ACS Appl. Energy Mater. 2023, 6, 10542–10553. 10.1021/acsaem.3c01670.

[ref25] Hutchison P.; Smith L. E.; Rooney C. L.; Wang H.; Hammes-Schiffer S. Proton-Coupled Electron Transfer Mechanisms for CO2 Reduction to Methanol Catalyzed by Surface-Immobilized Cobalt Phthalocyanine. J. Am. Chem. Soc. 2024, 146, 20230–20240. 10.1021/jacs.4c05444. 38984971

[ref26] Hong Y. H.; Jia X.; Stewart-Jones E.; Kumar A.; Wedal J. C.; Alvarez-Hernandez J. L.; Donley C. L.; Gang A.; Gibson N. J.; Hazari N.; Houck M.; Jeon S.; Kim J.; Koh H.; Mayer J. M.; Mercado B. Q.; Nedzbala H. S.; Piekut N.; Quist C.; Stach E.; Zhang Y. Photoelectrocatalytic reduction of CO2 to formate using immobilized molecular manganese catalysts on oxidized porous silicon. Chem. 2025, 102462 10.1016/j.chempr.2025.102462. PMC12396503 40894117

[ref27] Saund S. S.; Dabak-Wakankar A.; Gish M. K.; Neale N. R. Silicon nanocrystal hybrid photocatalysts as models to understand solar fuels producing assemblies. Sustain. Energy Fuels 2024, 8, 403–409. 10.1039/D3SE01512B.

[ref28] Wheeler L. M.; Anderson N. C.; Palomaki P. K. B.; Blackburn J. L.; Johnson J. C.; Neale N. R. Silyl Radical Abstraction in the Functionalization of Plasma-Synthesized Silicon Nanocrystals. Chem. Mater. 2015, 27, 6869–6878. 10.1021/acs.chemmater.5b03309.

[ref29] Carroll G. M.; Limpens R.; Neale N. R. Tuning Confinement in Colloidal Silicon Nanocrystals with Saturated Surface Ligands. Nano Lett. 2018, 18, 3118–3124. 10.1021/acs.nanolett.8b00680. 29659285

[ref30] Hanrahan M. P.; Fought E. L.; Windus T. L.; Wheeler L. M.; Anderson N. C.; Neale N. R.; Rossini A. J. Characterization of Silicon Nanocrystal Surfaces by Multidimensional Solid-State NMR Spectroscopy. Chem. Mater. 2017, 29, 10339–10351. 10.1021/acs.chemmater.7b03306.

[ref31] Hanrahan M. P.; Chen Y.; Blome-Fernández R.; Stein J. L.; Pach G. F.; Adamson M. A. S.; Neale N. R.; Cossairt B. M.; Vela J.; Rossini A. J. Probing the Surface Structure of Semiconductor Nanoparticles by DNP SENS with Dielectric Support Materials. J. Am. Chem. Soc. 2019, 141, 15532–15546. 10.1021/jacs.9b05509. 31456398

[ref32] Lee B. G.; Luo J. W.; Neale N. R.; Beard M. C.; Hiller D.; Zacharias M.; Stradins P.; Zunger A. Quasi-Direct Optical Transitions in Silicon Nanocrystals with Intensity Exceeding the Bulk. Nano Lett. 2016, 16, 1583–1589. 10.1021/acs.nanolett.5b04256. 26898670

[ref33] Muller A. V.; Ahmad S.; Sirlin J. T.; Ertem M. Z.; Polyansky D. E.; Grills D. C.; Meyer G. J.; Sampaio R. N.; Concepcion J. J. Reduction of CO to Methanol with Recyclable Organic Hydrides. J. Am. Chem. Soc. 2024, 146, 10524–10536. 10.1021/jacs.3c14605. 38507247

[ref34] Rose M. J. Semiconductor Band Structure, Symmetry, and Molecular Interface Hybridization for the Chemist. J. Am. Chem. Soc. 2024, 146, 5735–5748. 10.1021/jacs.3c07740. 38407043

[ref35] Jackson M. N.; Oh S.; Kaminsky C. J.; Chu S. B.; Zhang G.; Miller J. T.; Surendranath Y. Strong Electronic Coupling of Molecular Sites to Graphitic Electrodes via Pyrazine Conjugation. J. Am. Chem. Soc. 2018, 140, 1004–1010. 10.1021/jacs.7b10723. 29216428

[ref36] Jackson M. N.; Kaminsky C. J.; Oh S.; Melville J. F.; Surendranath Y. Graphite Conjugation Eliminates Redox Intermediates in Molecular Electrocatalysis. J. Am. Chem. Soc. 2019, 141, 14160–14167. 10.1021/jacs.9b04981. 31353897 PMC6748662

[ref37] Jackson M. N.; Surendranath Y. Molecular Control of Heterogeneous Electrocatalysis through Graphite Conjugation. Acc. Chem. Res. 2019, 52, 3432–3441. 10.1021/acs.accounts.9b00439. 31714746

[ref38] Hutchison P.; Kaminsky C. J.; Surendranath Y.; Hammes-Schiffer S. Concerted Proton-Coupled Electron Transfer to a Graphite Adsorbed Metalloporphyrin Occurs by Band to Bond Electron Redistribution. ACS Cent. Sci. 2023, 9, 927–936. 10.1021/acscentsci.3c00186. 37252356 PMC10214502

[ref39] Kayanuma Y. Quantum-size effects of interacting electrons and holes in semiconductor microcrystals with spherical shape. Phys. Rev. B: Condens. Matter 1988, 38, 9797–9805. 10.1103/PhysRevB.38.9797. 9945800

[ref40] Delerue C.; Allan G.; Lannoo M. Theoretical aspects of the luminescence of porous silicon. Phys. Rev. B: Condens. Matter 1993, 48, 11024–11036. 10.1103/PhysRevB.48.11024. 10007407

[ref41] Luo J.-W.; Stradins P.; Zunger A. Matrix-embedded silicon quantum dots for photovoltaic applications: a theoretical study of critical factors. Energy Environ. Sci. 2011, 4, 2546–2557. 10.1039/c1ee01026c.

[ref42] Smieja J. M.; Kubiak C. P. Re(bipy-tBu)(CO)3Cl–improved Catalytic Activity for Reduction of Carbon Dioxide: IR-Spectroelectrochemical and Mechanistic Studies. Inorg. Chem. 2010, 49, 9283–9289. 10.1021/ic1008363. 20845978

[ref43] Carroll G. M.; Limpens R.; Pach G. F.; Zhai Y.; Beard M. C.; Miller E. M.; Neale N. R. Suppressing Auger Recombination in Multiply Excited Colloidal Silicon Nanocrystals with Ligand-Induced Hole Traps. J. Phys. Chem. C 2021, 125, 2565–2574. 10.1021/acs.jpcc.0c11388.

[ref44] Limpens R.; Pach G. F.; Mulder D. W.; Neale N. R. Size-Dependent Asymmetric Auger Interactions in Plasma-Produced n- and p-Type-Doped Silicon Nanocrystals. J. Phys. Chem. C 2019, 123, 5782–5789. 10.1021/acs.jpcc.9b00223.

[ref45] Limpens R.; Pach G. F.; Neale N. R. Nonthermal Plasma-Synthesized Phosphorus–Boron co-Doped Si Nanocrystals: A New Approach to Nontoxic NIR-Emitters. Chem. Mater. 2019, 31, 4426–4435. 10.1021/acs.chemmater.9b00810.

[ref46] Pach G. F.; Carroll G. M.; Zhang H.; Neale N. R. Modulating donor–acceptor transition energies in phosphorus–boron co-doped silicon nanocrystals via X- and L-type ligands. Faraday Discuss. 2020, 222, 201–216. 10.1039/C9FD00106A. 32108841

[ref47] Wang K.; Cline R. P.; Schwan J.; Strain J. M.; Roberts S. T.; Mangolini L.; Eaves J. D.; Tang M. L. Efficient photon upconversion enabled by strong coupling between silicon quantum dots and anthracene. Nat. Chem. 2023, 15, 1172–1178. 10.1038/s41557-023-01225-x. 37308710

[ref48] Li H.; Wu Z.; Zhou T.; Sellinger A.; Lusk M. T. Tailoring the optical gap of silicon quantum dots without changing their size. Phys. Chem. Chem. Phys. 2014, 16, 19275–19281. 10.1039/C4CP03042G. 25098607

[ref49] Zhou T.; Anderson R. T.; Li H.; Bell J.; Yang Y.; Gorman B. P.; Pylypenko S.; Lusk M. T.; Sellinger A. Bandgap Tuning of Silicon Quantum Dots by Surface Functionalization with Conjugated Organic Groups. Nano Lett. 2015, 15, 3657–3663. 10.1021/nl504051x. 25971956

[ref50] Choate J. C.; Silva I. Jr.; Hsu P. C.; Tran K.; Marinescu S. C. The Positional Effect of an Immobilized Re Tricarbonyl Catalyst for CO2 Reduction. ACS Appl. Mater. Interfaces 2024, 16, 50534–50549. 10.1021/acsami.4c05536. 39255361

[ref51] Rehm J. M.; McLendon G. L.; Fauchet P. M. Conduction and Valence Band Edges of Porous Silicon Determined by Electron Transfer. J. Am. Chem. Soc. 1996, 118, 4490–4491. 10.1021/ja9538795.

[ref52] Kumar A.; Sun S.-S.; Lees A. J. Photophysics and Photochemistry of Organometallic Rhenium Diimine Complexes. Top. Organomet. Chem. 2010, 29, 1–35. 10.1007/3418_2009_2.

[ref53] Lian S.; Kodaimati M. S.; Dolzhnikov D. S.; Calzada R.; Weiss E. A. Powering a CO2 Reduction Catalyst with Visible Light through Multiple Sub-picosecond Electron Transfers from a Quantum Dot. J. Am. Chem. Soc. 2017, 139, 8931–8938. 10.1021/jacs.7b03134. 28608682

[ref54] Lian S.; Kodaimati M. S.; Weiss E. A. Photocatalytically Active Superstructures of Quantum Dots and Iron Porphyrins for Reduction of CO2 to CO in Water. ACS Nano 2018, 12, 568–575. 10.1021/acsnano.7b07377. 29298382

[ref55] Eliasson N.; Rimgard B. P.; Castner A.; Tai C.-W.; Ott S.; Tian H.; Hammarström L. Ultrafast Dynamics in Cu-Deficient CuInS2 Quantum Dots: Sub-Bandgap Transitions and Self-Assembled Molecular Catalysts. J. Phys. Chem. C 2021, 125, 14751–14764. 10.1021/acs.jpcc.1c02468.

[ref56] Martinez M. S.; Nolen M. A.; Pompetti N. F.; Richter L. J.; Farberow C. A.; Johnson J. C.; Beard M. C. Controlling Electronic Coupling of Acene Chromophores on Quantum Dot Surfaces through Variable-Concentration Ligand Exchange. ACS Nano 2023, 17, 14916–14929. 10.1021/acsnano.3c03498. 37494884 PMC10416565

[ref57] Hawecker J.; Lehn J. M.; Ziessel R. Photochemical and Electrochemical Reduction of Carbon Dioxide to Carbon Monoxide Mediated by (2,2′-Bipyridine)tricarbonylchlororhenium(I) and Related Complexes as Homogeneous Catalysts. Helv. Chim. Acta 1986, 69, 1990–2012. 10.1002/hlca.19860690824.

