# Supplementary material for: Design Strategies for Coupling CO2 Reduction Molecular Electrocatalysts to Silicon Photocathodes
Source: ACS Mater Au. 2025 Apr 14;5(3):569–79. doi: 10.1021/acsmaterialsau.5c00010 (PMC12082352; doi:10.1021/acsmaterialsau.5c00010)
Supplement: Supplementary file 1 — mg5c00010_si_001.pdf [file mg5c00010_si_001.pdf]

Supporting Information for:

Design Strategies for Coupling CO_2 Reduction Molecular Electrocatalysts to Silicon Photocathodes

Simran S. Saund,[a] Melissa K. Gish,[a] Jeremiah Choate,[b] Trung Le,[a] Smaranda C. Marinescu,[b] and Nathan R. Neale*[a,c]

[a]Chemistry and Nanoscience Center, National Renewable Energy Laboratory, Golden, Colorado 80401, United States

[b]Department of Chemistry, University of Southern California, Los Angeles, California 90089, United States

[c]Renewable and Sustainable Energy Institute, University of Colorado Boulder, Boulder, Colorado 80309, United States

Table of Contents

I. **General materials and methods** S2
II. **B:Si-CH_3CN preparation** S2
III. **B:Si-OCH_2[Re]** S2
IV. **B:Si-C_{Ar}[Re]** S3
V. **FTIR** S3
VI. **Electrochemistry** S3
VII. **UV-visible spectroscopy** S3
VIII. **Spectroelectrochemistry** S3
IX. **Spectroelectrochemical analysis** S4
X. **Figure S0 spectroelectrochemistry of B:Si-CH_3CN, B:Si-OCH_2[Re], and B:Si-C_{Ar}[Re]** S5
XI. **Photoluminescence** S5
XII. **Transient absorption spectroscopy** S5
XIII. **Photocatalysis** S5
XIV. **Computational details** S5
XV. **Figure S1 DRIFTS spectra of 3.0 – 3.9 nm ^{i}Si-OCH_2[Re]** S7
XVI. **Figure S2 PL and UV-Vis spectra of 3.0 – 3.9 nm ^{i}Si-OCH_2[Re]** S7
XVII. **Figure S3 transient absorption spectra of 3.0 and 3.9 nm ^{i}Si-C_{12} and ^{i}Si-OCH_2[Re]** S8
XVIII. **Table S1 transient decay kinetics** S8
XIX. **Surface [Re] quantification for 3.0 – 3.9 nm ^{i}Si-OCH_2[Re]** S9
XX. **Figure S4 liquid FTIR spectra for tethered [Re] determination** S9
XXI. **Figure S5 catalytic product analysis** S10
XXII. **Figure S6 Schematic depiction of the local surface environment** S10
XXIII. **Figure S7 DFT predicted bandgap trends** S11
XXIV. **Scheme S1 Si-[Re] tethers and [Re] precursor compounds** S11
XXV. **Figure S8 computed energy levels of 1.5 and 2.0 nm naked ^{i}Si and B:Si** S12
XXVI. **Figure S9 tether dependent computed energy levels of 1.5 nm B:Si-[Re]** S13

XXVII. Figure S10 DRIFTS of B:Si-CH_3CN, B:Si-OCH_2[Re], and B:Si-C_{Ar}[Re] S14
XXVIII. Figure S11 FTIR spectra of B:Si-C_{Ar}[Re], [Re]N_2 (pristine), and [Re]N_2 (degraded) S14
XXIX. CV data analysis S15
XXX. Figure S12 cyclic voltammograms of B:Si-CH_3CN, B:Si-OCH_2[Re], and B:Si-C_{Ar}[Re] S16
XXXI. Figure S13 precycled voltammograms of B:Si-CH_3CN, B:Si-OCH_2[Re], and B:Si-C_{Ar}[Re] S17
XXXII. Figure S14 transient spectra of B:Si species and mixtures with [Re] S18
XXXIII. Figure S15 Photocatalytic CO_2RR of B:Si species S19
XXXIV. References S19

Experimental

General materials and methods

Solvents and 2,2,2-tetrafluoroethanol (TFE) were purchased from Sigma Aldrich and dried using standard methods. Tetrabutylammonium hexafluorophosphate ($TBAPF_6$), 1,3-Dimethyl-2-phenyl-2,3-dihydro-1H-benzo[d]imidazole (BIH), and 4-methyl-2,2'-bipyridyl-4'-carboxaldehyde (mcabpy) were purchased from Sigma and dried over P_2O_5. Rhenium pentacarbonyl bromide was purchased from Strem and Rhenium pentacarbonyl chloride was purchased from ThermoFisher Scientific, both were used as received. All work was performed in an argon filled glovebox unless otherwise stated. [Re(mcabpy)$(CO)_3$Br],[1] [Re(bpy)$(CO)_3$Br],[2] ^{i}Si-OCH_2[Re],[1] ^{i}Si-C_{12}[1, 3] and B:Si[4] were prepared as previously reported, with ^{i}Si NP size determined from the emission energy maximum of ^{i}Si-C_{12} applied to a sizing curve[5] and B:Si NP size determined from Scherrer analysis of the (111) diffraction peak.[4] [Re($bpyN_2$)$(CO)_3$Br]BF_4 ($bpyN_2$ = 2,2'-bipyridyl-4-diazonium) was prepared as previously reported with the 2,2'-bipyridyl-4,4'-diamine precursor ligand substituted for 2,2'-bipyridyl-4-amine.[6] Intrinsic and boron doped nanocrystals were grown by plasma-enhanced chemical vapor deposition as reported previously.[3-5] ITO coated glass slides (350.0 ± 30.0 nm, 3–5 $\Omega \cdot sq^{-1}$) were purchased from MSE and sonicated in isopropanol then rinsed with methanol after cutting to size. Cleaned ITO substrates were dried in a 150 °C oven overnight and then stored in an argon-filled glovebox.

B:Si-CH_3CN preparation

In a representative reaction, 11.9 mg 3.9 nm B:Si NC (est. 0.31 μmols) and 3.12 mL CH_3CN were charged into a scintillation vial. The reaction was sealed and then sonicated for 3 h yielding a dark coffee-colored colloid of 100 μM B:Si-CH_3CN. The colloidal sample was returned to the glovebox and filtered through an 0.7 μm glass microfiber plug.

B:Si-OCH_2[Re] (1:1)

In a representative reaction, 11.0 mg 3.9 nm B:Si (est. 0.29 μmols) was charged into a scintillation vial with 288 μL 1 mM [Re(mcabpy)$(CO)_3$Br] in CH_3CN and 5.47 mL fresh CH_3CN. The resulting mixture was sealed and sonicated for 3 h yielding a 100 μM colloid of 100 μM B:Si-OCH_2[Re]. The sample was returned to the glovebox and filtered through a 0.7 μm glass microfiber plug.

B:Si-C_{Ar}[Re]

In a representative reaction, 41.1 mg 3.9 nm B:Si (est. 1.08 µmols) was charged into a scintillation vial with 1.08 mL of 1 mM [Re(dabpy)$(CO)_3$Cl]BF_4 in CH_3CN. The vessel was sealed and sonicated for 1 h yielding a dark, viscous slurry. The reaction was returned to the glovebox and 9.68 mL of fresh CH_3CN were added. The vial was resealed then sonicated for another 2 h yielding a dark coffee colored colloid of 100 µM B:Si-C_{Ar}[Re]. The product was returned to the glovebox and filtered through a 0.7 µm glass microfiber plug.

FTIR

FTIR measurements were taken on a Bruker Alpha II FTIR spectrometer housed in an argon-filled glovebox and fitted with either a diffuse reflectance Fourier transform spectroscopy (DRIFTS) module, diamond attenuated total reflectance (ATR) module, or transmission module. DRIFTS samples were acquired by either drop-casting an analyte onto an Au-coated reflective silicon wafer substrate, or directly depositing the powder analyte onto the same substrate. ATR measurements were acquired by directly clamping the powder sample onto the ATR prism. Transmission liquid FTIR measurements were acquired using a 1 mm path length KBr liquid IR cell.

Electrochemistry

All electrochemical measurements were taken in an argon-filled glovebox on a two channel Biologic SP-300 potentiostat. Thin film CVs were taken in CH_3CN with 100 mM $TBAPF_6$ as supporting electrolyte. 0.3 cm diameter glassy carbon electrodes (GCE) were prepared by polishing on a 0.05 µm alumina slurry coated wet polishing pad, then rinsed with water and methanol. Sonication was avoided to protect the glassy carbon – metal pin connection. Thin films were prepared by dipping the GCE in a NC solution for ~1 s, then inverting and removing excess solution with a kimwipe. CVs were taken in a three-electrode setup with a platinum wire counter electrode and a silver pseudo reference electrode chambered with 100 mM $TBAPF_6$ in CH_3CN and separated from bulk electrolyte by a CoralPor® frit. Solution resistance was measured by impedance and corrected for *in situ*. Ferrocene was added at the end of each experiment batch to calibrate the pseudo reference electrode.

UV-Vis spectroscopy

Steady state UV-vis spectra in the absence of electrochemical influence were collected on samples in a 2 mm path length quartz cuvette with a CARY-5000 spectrophotometer equipped with a Xe flash bulb.

Spectroelectrochemistry

Spectroelectrochemistry (SEC) was performed on thin film samples deposited on ITO coated (350.0 ± 30.0 nm, 3 – 5 $\Omega \cdot sq^{-1}$) glass slides using the same potentiostat setup described above. White light was supplied to the sample by an Ocean Optics 20 W tungsten halogen lamp delivered into the glovebox by fiberoptic with the transmitted light collected by fiberoptic and delivered to an OceanFX UV-Vis and NIR detector. Films were formed by "painting" the analyte solution onto the slide with a glass pipet until a transparent film had formed. Electrical contact was made to the slide using copper tape (adhered well above the electrolyte solution and separated from the deposited analyte film). The measurements were taken in a

1.7 mm path length quartz cuvette with a larger well in the top half. A three-electrode configuration was employed, with a Pt wire counter electrode and chambered Ag pseudo reference electrode. Prior to SEC acquisition, the reference electrode was calibrated in a solution of ferrocene in methanol with 100 mM $TBAPF_6$ as supporting electrolyte. The measurements were acquired in CH_3CN with 100 mM $TBAPF_6$ as supporting electrolyte. Approximately 3-min duration controlled potential electrolysis was performed at various potentials and spectra were collected every 30 s with ~7 s signal averaging.

Spectroelectrochemical analysis

We conduct a series of UV-Vis spectroelectrochemical (SEC) experiments on thin films of B:Si-CH_3CN or B:Si-OCH_2[Re] on ITO coated glass substrates (Fig. S0). At an applied potential of -2.5 V vs. $Fc^{+/0}$, a species with peaks at ~510 nm and ~700 nm grows in. In our previous report on the ^{i}Si-OCH_2[Re] system, we assigned the 510 nm feature as a bpy-centered radical anion arising from direct reduction of the surface tethered [Re] complex.[1] However, the presence of a similar feature in naked B:Si-CH_3CN suggests that the feature is instead Si centered. The lack of SEC response in dodecyl-terminated ^{i}Si-C_{12} is likely due to the insulating nature of the dodecyl ligand sphere, hampering electron transfer into the Si NC, consistent with our observation that the dodecyl molecular coating shuts down ionic and electrical transport when Si NCs are used as the active anode material for lithium-ion batteries.[7] The lack of any auxiliary ligand for B:Si-CH_3CN enhances electron/electrolyte mobility through the film, allowing SEC signals from the electrochemically doped B:Si species to be observed. Upon applying a potential of 0.5 V vs. $Fc^{+/0}$ to the same film, a large bleach peaking at 410 nm grows in and the entire trace shifts below 0. The same experiment is performed on films of B:Si-OCH_2[Re] and B:Si-C_{Ar}[Re]. B:Si-OCH_2[Re] behaves similarly. Generally, a peak around 510 nm is again observed on application of -2.5 V vs. $Fc^{+/0}$, but much sharper than that for films of B:Si-CH_3CN. However, at an applied potential of 0.5 V vs. $Fc^{+/0}$, the 510 nm peak is diminished slightly while a small but broad increase in absorbance is observed between 620 nm and the edge of our detector. B:Si-C_{Ar}[Re] films appear intermediate between B:Si-CH_3CN and B:Si-OCH_2[Re] in overall magnitudes, but the overall peak shapes more closely match that of B:Si-OCH_2[Re]. However, variance in film thickness and orientation may be responsible for amplitude differences. Generally, the presence of surface [Re] appears to suppress the 410 nm bleach observed upon positive polarization of the working electrode (Fig. 6, Fig. S11). This observation is consistent with CV data that suggests suppression of the broad oxidation > –500 mV vs. $Fc^{+/0}$ by tethered Re.

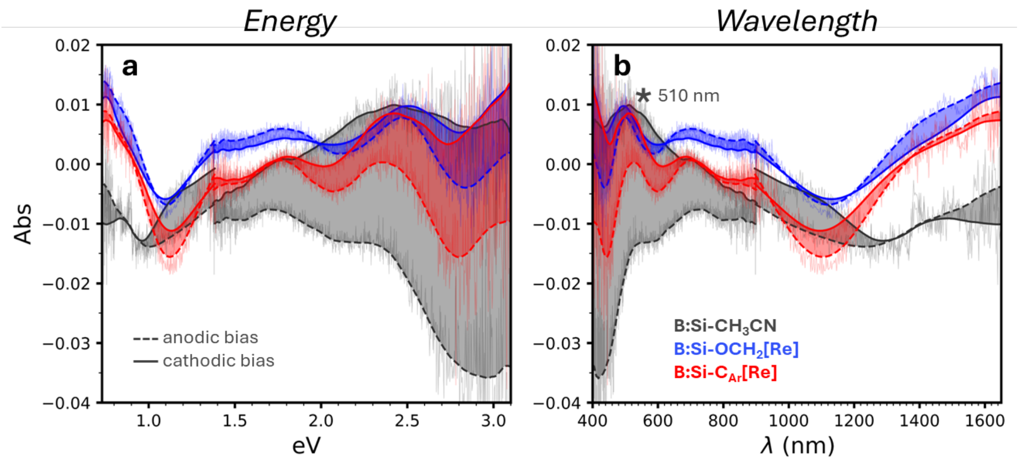

Fig. S0 Spectroelectrochemical plots of B:Si-CH_3CN (grey), B:Si-OCH_2[Re] (blue), and B:Si-C_{Ar}[Re] (red) thin films on ITO during anodic (~ 0.5 V vs. $Fc^{+/0}$, dashed lines) or cathodic (~ –2.5 V vs. $Fc^{+/0}$ solid lines) biasing.

Photoluminescence

Emission spectra were acquired on solution phase samples excited by a 1.4 A ThorLabs LED. The excitation source was filtered through a 450 nm shortpass filter and 410 bandpass filter and delivered to the sample by fiberoptic. The emitted light was collected at 0° from the excitation source and filtered through a 450 nm lowpass filter then delivered to an OceanFX UV-vis detector and NIRQuest near IR detector.

Transient Absorption Spectroscopy

Transient absorption data were collected using a Ti:Sapphire regenerative amplifier with an 800 nm fundamental (1 kHz, 90 fs pulse width) (Coherent Astrella). The beam is split at the outset to create the pump and probe beam paths. The 400 nm pump (1 mW) is generated in an optical parametric amplifier (TOPAS, Light Conversion) and chopped at 500 Hz to modulate the pump on and off. The probe pulse travels through a multi-pass mechanical delay stage and focused through a thin sapphire crystal to generate a white light supercontinuum (λprobe = 440-850 nm). The pump and the probe are focused and spatially overlapped at the sample. A small portion of the probe is picked off before the sample to act as a reference to maximize the signal-to-noise ratio. Changes in the probe spectrum were monitored by a fiber-coupled multichannel spectrometer with a CMOS sensor. Data were collected and chirp corrected using Helios and Surface Xplorer programs (Ultrafast Systems), respectively. Data were analyzed in Origin (OriginLabs).

Photocatalysis of ^{i}Si-[Re] samples

Photocatalysis was performed in 3.7 mL (dram) vials sealed with a silicone septum. The vials were filled with 1 mL saturated BIH in THF (measured to be 200 mM if fully dissolved, and sonicated for 20 minutes prior to use), 15 µL of TFE (102 mM), 40 µL of 50 µM ^{i}Si-[Re] colloid in toluene, and 960 µL fresh THF. Control samples containing either ^{i}Si-C_{12} and [Re(bpy)$(CO)_3$Br] or just [Re(bpy)$(CO)_3$Br] were also prepared using the same amounts for TFE and THF described above, but adding 40 µL of 50 µM ^{i}Si-C_{12}, 10 µL of 200

μM [Re(bpy)$(CO)_3$Br], and 950 μL fresh THF or 10 μL [Re(bpy)$(CO)_3$Br] and 990 μL of fresh THF (respectively). In all cases, the total solution volume was 2.015 mL, 0.99 μM catalyst, 0.99 μM ^{i}Si-C_{12} when relevant, saturated BIH (measured to be 99.3 mM in the final solution if fully dissolved), and 102 mM TFE. The vial was removed from the glovebox and purged with CO_2 for 20 minutes via a 22 gauge sideport needle. The sample was then photolyzed in a HepatoChem PhotoRedOx Box™ photo reactor for 20 h. Gaseous products were determined by injection of 200 μL headspace into an SRI 8610C gas chromatograph (GC) in the Multiple Gas Analyzer #5 configuration. The headspace sample was directly injected onto a 1.5 m 5 Å molecular sieve column and products were analyzed with a thermal conductivity detector (TCD) and in-line methanizer equipped flame ionization detector (FID). Ar carrier gas was used to enhance TCD sensitivity for H_2, while CO and CH_4 were analyzed by FID. Products were quantitated using a calibration curve generated from standard injections of a custom gas blend supplied by GASCO. All photocatalysis measurements were performed in triplicate with error bars representing the standard deviation of peak areas between the three runs.

Computational details

DFT modeling was performed with the ORCA 5.0.3[8-10] software suite and parallelized with OpenMPI. Libraries including libint2,[11] libXC,[12] and XCFun[13] were used during calculations or for the ORCA build. Geometry optimizations were performed at the M06L level of theory, with SVP applied to all H, C, and Si atoms and TZVP applied to all other atoms.[14] Zero order relativistic approximations were implemented for Re containing models and the segmented all-electron relativistically contracted form of the TZVP basis set was applied to the Re atom.[15] The SARC/J auxiliary basis set was applied to all models containing Re while def2/J was applied to all others.[15-16] Atom-pairwise dispersion corrections with the zero-damping scheme (D30) were employed and all calculations were performed in an implicit solvation environment using the conductor-like polarizable continuum model (CPCM) with standard CH_3CN parameters.[17] The models varied in size from ~150 atoms to ~370 atoms and thus frequency calculations proved prohibitively expensive. Orbitals were visualized using ChemCraft. Atomic contributions to molecular orbitals were determined from the Mulliken population analysis output. In short, atomic orbital contributions were summed for each atom type then represented as a percentage of the total molecular orbital composition. [Re] complex contribution was determined as the sum total of C, N, O, Br, and Re contributions, Si NC contribution was determined as the sum total individual Si atom contributions and B trap state contributions were determined as the sum total of individual B contributions. H contributions were ignored.

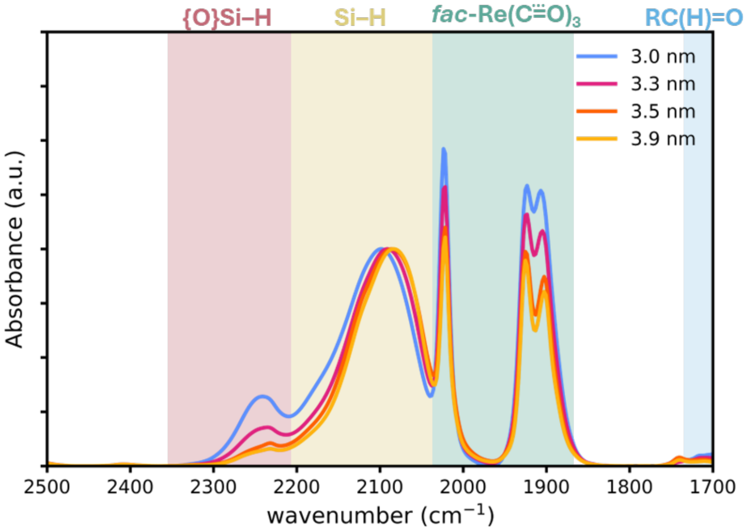

Fig. S1 DRIFTS spectra of 3.0 – 3.9 nm ^{i}Si-OCH$_2$[Re] showing the back-bonded (O)*Si–H (red shading), *Si–H (yellow shading), *fac*-Re(CO)$_3$ (green shading), and aldehyde (blue shading) regions.

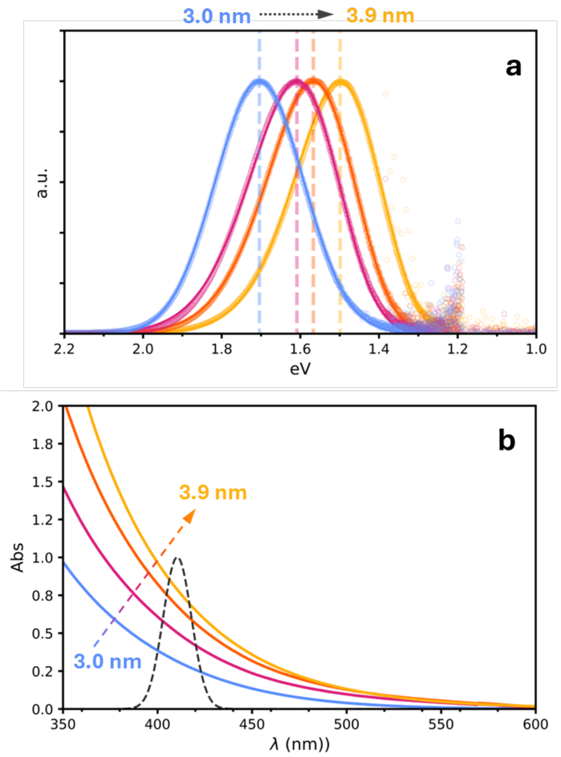

Fig. S2 a) PL spectra of 3.0 – 3.9 nm ^{i}Si-OCH$_2$[Re] in toluene. Each of the experimental data sets (colored circles) is overlayed with a Gaussian fit, used to determine PL maxima at 1.70(8), 1.62(1), 1.57(6), and 1.51(5) eV for 3.0, 3.3, 3.5, and 3.9 nm assemblies, respectively. b) Steady-state absorption spectra for 3.0 – 3.9 nm ^{i}Si-OCH$_2$[Re] assemblies (colored solid traces) and a normalized emission spectra of the 405 nm LED array used during photocatalysis (black dashed line). Spectra acquired on samples at 10 µM ^{i}Si-OCH$_2$[Re] in 1:4 toluene:THF with a 2 mm path length cuvette.

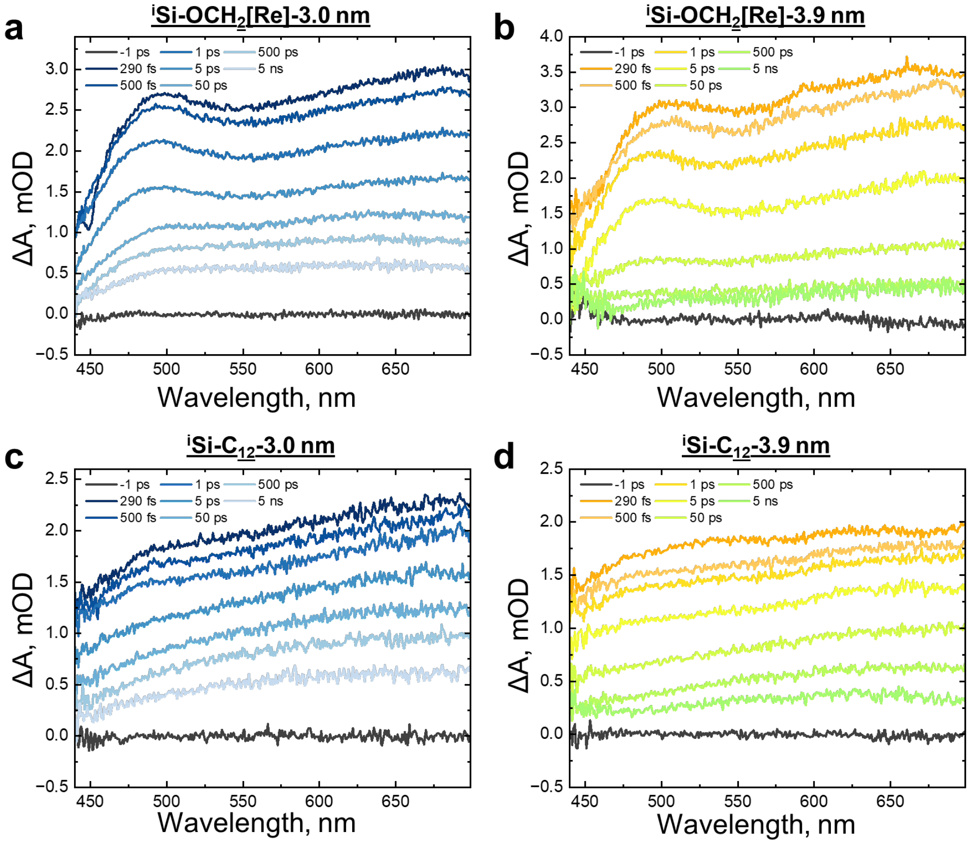

Fig. S3 Transient absorption spectra of a) 3.0 nm ⁱSi-OCH₂[Re], b) 3.9 nm ⁱSi-OCH₂[Re], c) 3.0 nm ⁱSi-C_{12}, or d) 3.9 nm ⁱSi-C_{12}. All spectra acquired after excitation at 400 nm, samples were prepared at 1 μM in a 1:4 mixture of toluene:THF under an Ar atmosphere.

	ⁱSi-C_{12}		iSi-OCH2[Re]	
	**3.0 nm**	**3.9 nm**	**3.0 nm**	**3.9 nm**
y_0	0.20 (±0.004)	0.13 (±0.004)	0.20 (±0.005)	0.07 (±0.004)
A_1	0.42 (±0.01)	0.39 (±0.01)	0.46 (±0.01)	0.44 (±0.01)
τ_1, ps	1.7 (±0.10)	1.9 (±0.12)	1.7 (±0.10)	2.3 (±0.15)
A_2	0.26 (±0.01)	0.31 (±0.01)	0.25 (±0.01)	0.33 (±0.01)
τ_2, ps	26 (±2.0)	37 (±2.6)	25 (±2.4)	39 (±3.2)
A_3	0.19 (±0.006)	0.19 (±0.008)	0.14 (±0.006)	0.14 (±0.01)
τ_3, ps	868 (±76)	635 (±59)	764 (±98)	580 (±89)

Table S1 Kinetic analysis of transient absorption spectra in Fig. 1.[3, 18]

Surface [Re] quantitation for 3.0 – 3.9 nm ⁱSi-OCH$_2$[Re]

[Re] loading was determined by FTIR. First, we characterize a standard series of 50, 100, 200, 500, and 1000 µM [Re(bpy)(CO)$_3$Br] in THF by transmission FTIR using a 1 mm path length liquid cell with KBr windows (Figure S4a, grey filled spectra). The peak heights of the 1899 cm^{-1} (out of phase symmetric) feature are used to generate a calibration curve (Figure S4b open circles and black dotted line) with the equation $y = 416.63*C_{[Re]} – 0.0026$ ($C_{[Re]}$ is concentration of [Re] in M). Finally, the peak heights of each assembly from 3.0 – 3.9 nm measured at a standard concentration of 50 µM in toluene (Figure S4b, colored dashed lines) are compared to the calibration curve to give $C_{[Re]}$ in each analyte sample (Figure S4b, inset table).

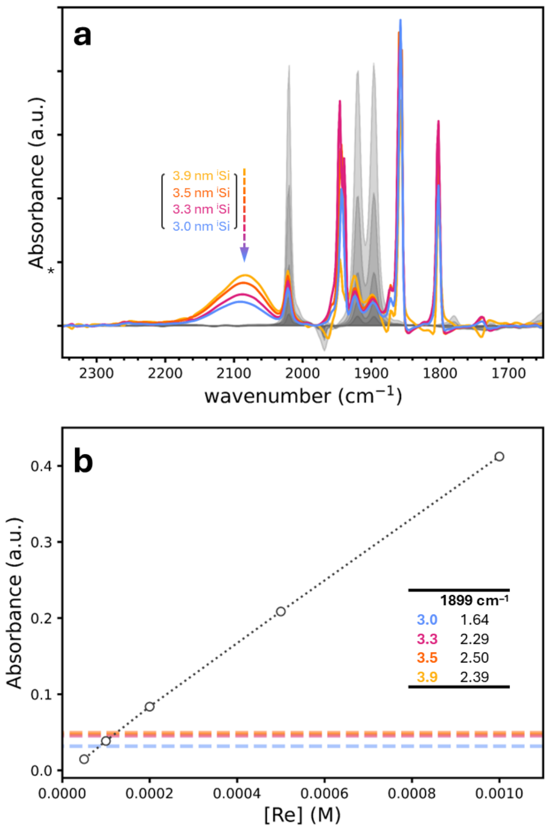

Fig. S4 a) FTIR spectra of 50 µM 3.0 – 3.9 nm ⁱSi-OCH$_2$[Re] in toluene (solid-colored traces) and a series of 50, 100, 200, 500, 1000 µM [Re(bpy)(CO)$_3$Br] in THF (grey filled traces). All spectra acquired in a 1 mm path length liquid IR cell with a KBr window. b) Calibration curve for [Re(bpy)(CO)$_3$Br] (black dashed line and black circles) determined from the 1899 cm^{-1} peak in plot (a). The formula of the calibration trend line is $y = 416.63*[Re] – 0.0026$ ([Re] in M). The colored horizontal lines correspond to ⁱSi-OCH$_2$[Re] peak intensities and the inset table shows the number of [Re] complexes per NC for 3.0 – 3.9 nm hybrid assemblies.

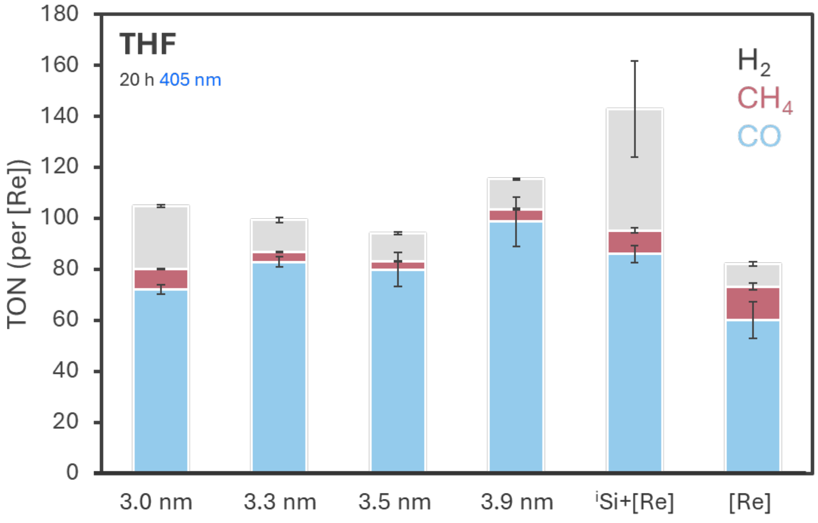

Fig. S5 Photocatalytic formation of H_2 (grey), CH_4 (red), and CO (blue) by ^{i}Si-OCH$_2$[Re] (3.0 – 3.9 nm series), 3.9 nm ^{i}Si+[Re], and [Re(bpy)(CO)$_3$Br]. Error bars representing the standard deviation between three separate photocatalytic measurements are included.

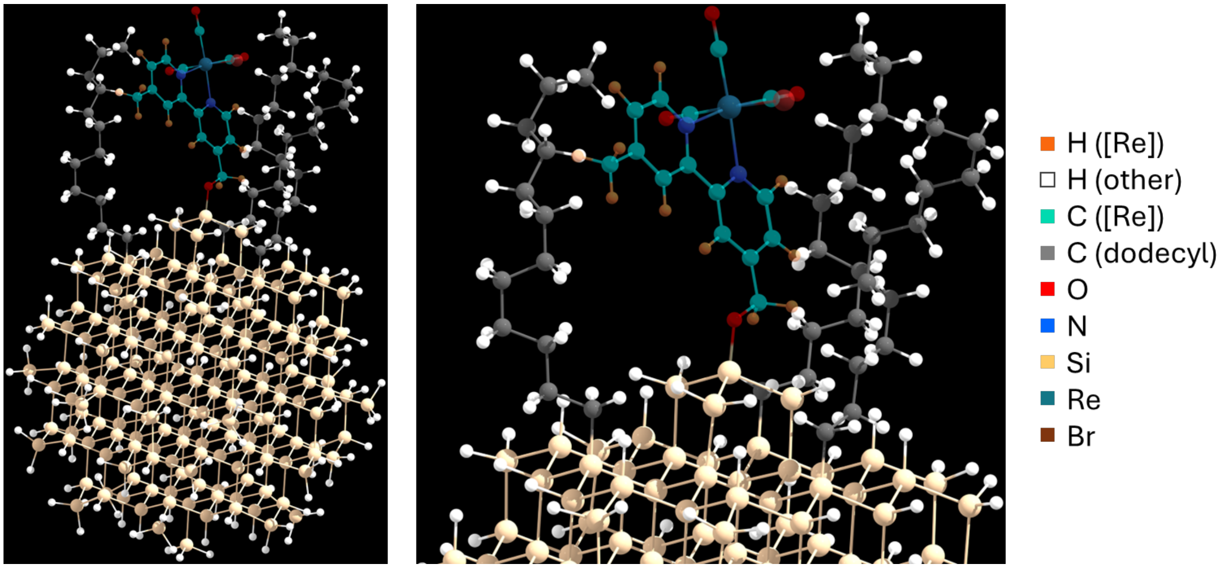

Fig. S6 Schematic depiction of the local surface environment around a tethered [Re] complex in ^{i}Si-OCH$_2$[Re]. The model represents a 2.0 nm diameter Si NC with a tethered [Re] catalyst optimized at the M06l/TZVP/SVP level of theory, 3 dodecyl ligands have been added over this ~1 nm^2 surface area post-optimization based on our prior quantification of 4-5 dodecyl ligands/nm^2 at the saturation limit[3] and are included with standard bond lengths and angles. For visual clarity, the C and H atoms present in the [Re] complex are colored uniquely (cyan and orange respectively) and all atoms comprising the [Re] complex are set to transparent.

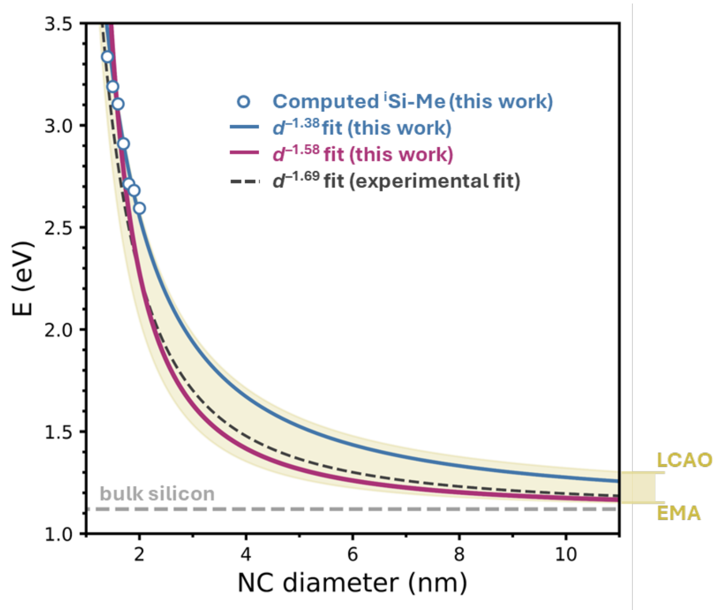

Fig. S7 The effects of quantum confinement on iSi NC band gaps. The DFT predicted results from this work are represented by blue circles and are fit with a power law trend according to a $d^{-1.38}$ relationship (blue line). Included is a second trend from DFT predictions in this work based on the band edge fits discussed in the main text (maroon line) and determined by $\Delta E = E_{CB} - E_{VB}$, where E_{CB} and E_{VB} are the conduction band and valence band energy trends represented by the orange dashed line and orange solid line (respectively) in Fig B. For comparison, the experimental band gap fit (black dashed line) is included.[5] The yellow shaded region represents the bounds between predicted band gap trends based on a linear combination of atomic orbitals (LCAO) approach or effective mass approximation approach.[19] The grey dashed line is the band gap of bulk silicon.

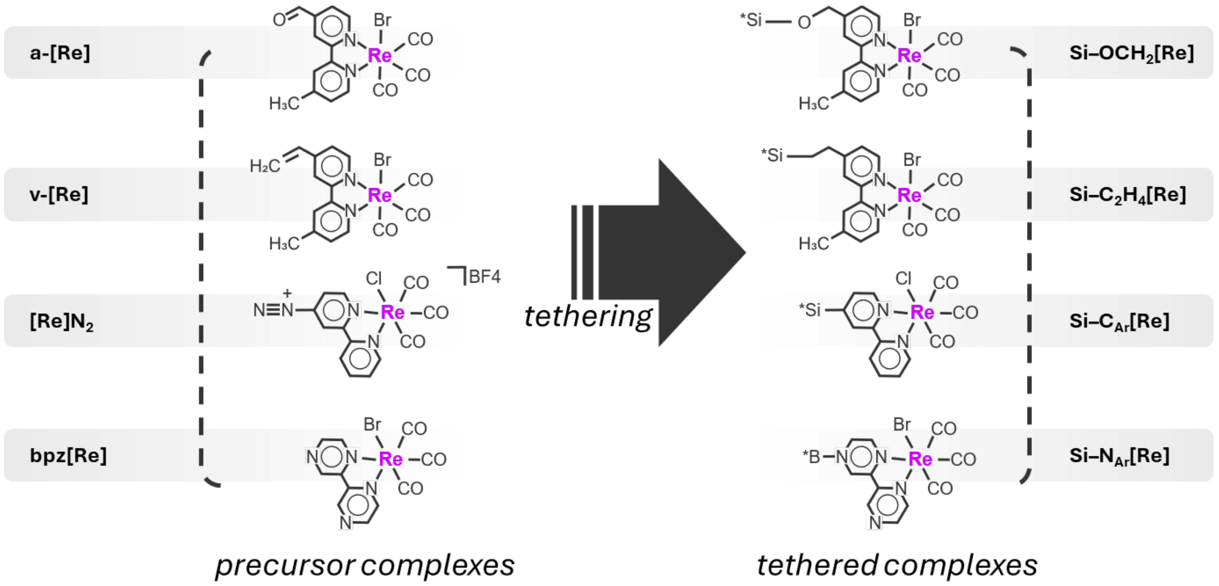

Scheme S1 Precursor [Re] complexes for the tethers considered computationally and the resulting linkages used for the computational models.

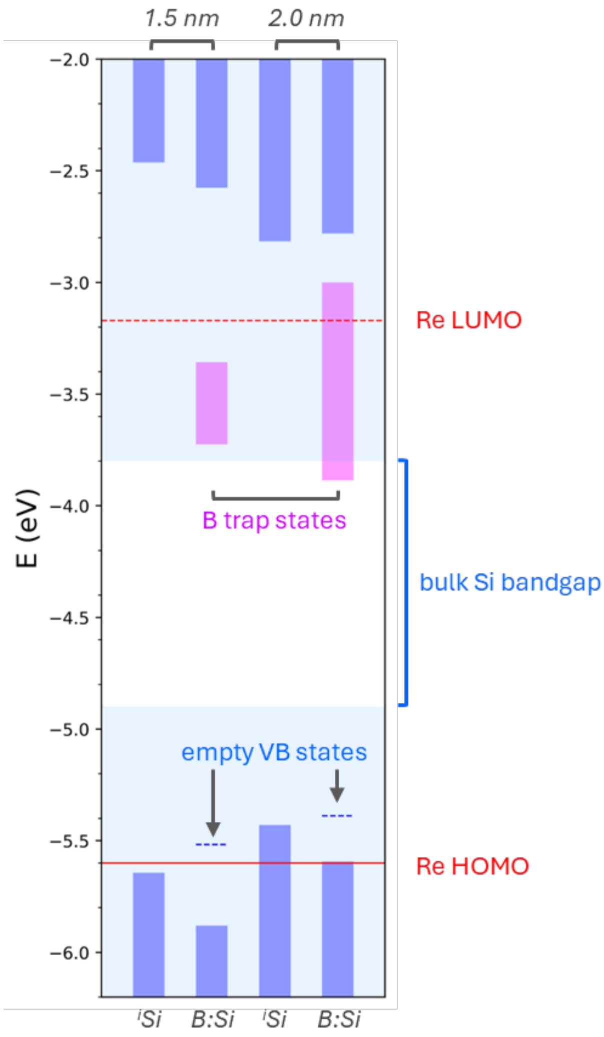

Fig. S8 DFT predicted energetics of 1.5 and 2.0 nm ^{i}Si and B:Si NC models. Blue shaded bars represent the Si NC bands, pink shaded bars represent B intragap states, and the light blue shaded regions represent the bulk Si band structure. The dashed blue lines represent empty valence band states in p-type B:Si. The unbound [Re(bpy)(CO)$_3$Br] HOMO and LUMO are shown as a solid or dashed red line, respectively, across the entire plot.

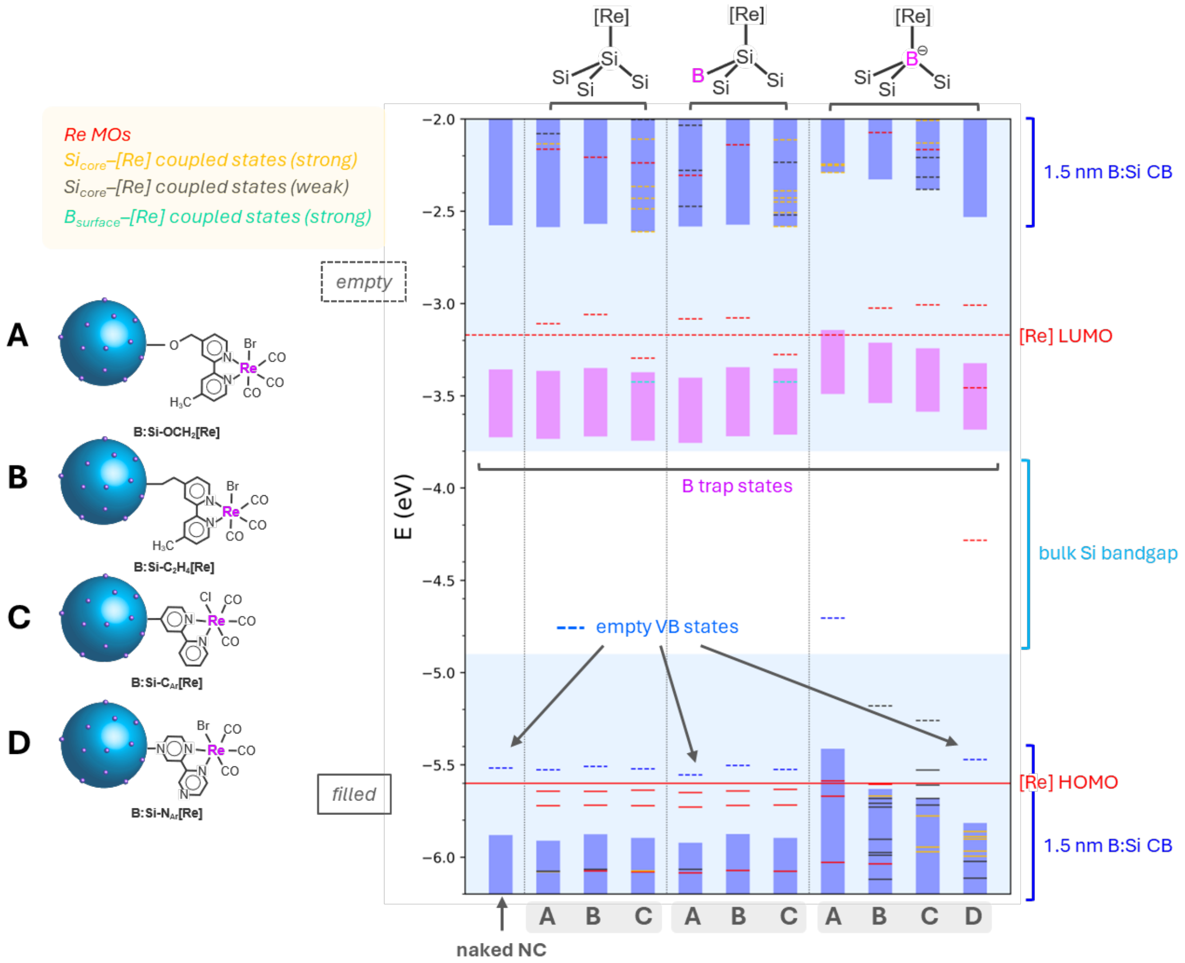

Fig. S9 DFT predicted energetics of 1.5 nm B:Si-[Re] models with the shown surface attachment chemistries (A-D). Blue shaded bars represent the B:Si NC bands, pink shaded bars represent B intragap states, and the light blue shaded regions represent the bulk Si band structure. Strongly coupled Si – [Re] states are shown as gold lines, [Re] centered orbitals are shown as red lines, slightly coupled states are shown as black or salmon lines for Si or [Re] dominant orbitals, respectively, and coupled B – [Re] states are shown as pink lines. Dashed lines represent empty orbitals and solid lines represent filled orbitals. The unbound [Re(bpy)(CO)$_3$Br] HOMO and LUMO are shown as solid and dashed red lines, respectively, across the entire plot.

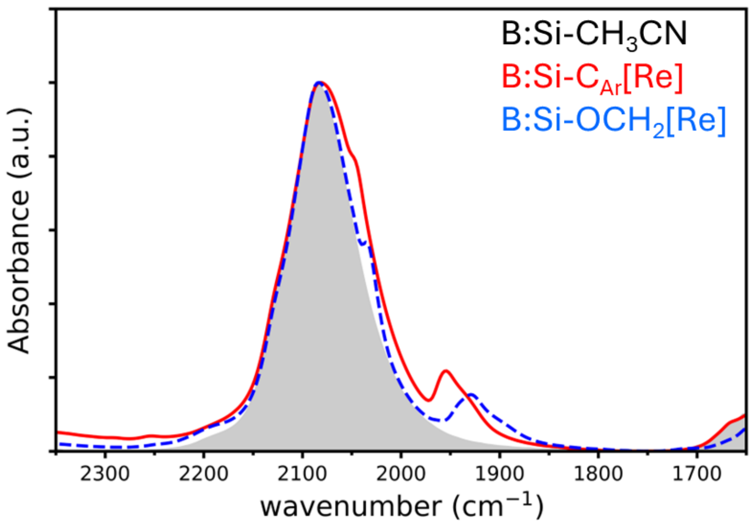

Fig. S10 DRIFTS spectra of B:Si-C_{Ar}[Re] (red solid line) and B:Si-OCH_2[Re] (blue dashed line) compared to B:Si-CH_3CN (grey shaded region).

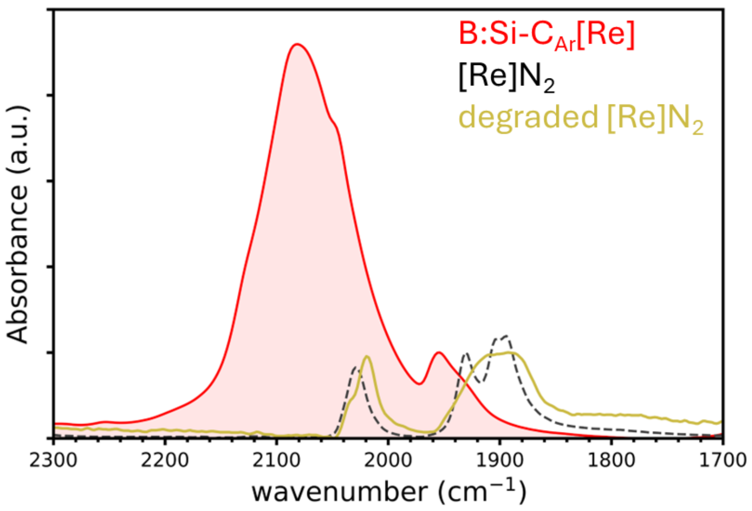

Fig. S11 DRIFTS spectra of B:Si-C_{Ar}[Re] (red solid line, red fill) and sonically degraded [Re]N_2 (yelow solid line) compared to a powder ATR spectrum of the pristine [Re]N_2 salt.

CV analysis

CVs of B:Si-CH_3CN show redox features in the –2.6 to 0.5 V vs. $Fc^{+/0}$ range (Fig. 6, Fig. S11). These redox features are not observed in CVs of dodecyl terminated ^{i}Si NC thin films (^{i}Si-C_{12}),[1] suggesting that the lack of insulating dodecyl ligands on B:Si-CH_3CN likely enhances both charge and ionic mobility through the thin film relative to ^{i}Si-C_{12}.[7] CVs of thin film B:Si-OCH_2[Re] show more distinct initial features in the cathodic region. Three waves ($E_{p,c}$ = –1.68, –1.84 and –2.14 V vs. $Fc^{+/0}$) are present on the first scan of B:Si-OCH_2[Re], with the first peak matching well with the first peak observed for B:Si-CH_3CN films (–1.66 V vs. $Fc^{+/0}$) and being gone by the 11th cycle. The two new features ($E_{p,c}$ = –1.84 and –2.14 V vs. $Fc^{+/0}$, $\Delta E_{p,c}$ = 0.30) match well the $Re^{I/0}$ and $Re^{0/-1}$ peak potentials for free [Re(bpy)$(CO)_3$Br] ($E_{p,c}$ = –1.84 and –2.16 V vs. $Fc^{+/0}$, $\Delta E_{p,c}$ = 0.32) and are preserved across 11 CV cycles. It's important to note that exact peak values for a feature shared between different samples is expected to shift slightly with factors such as film thickness which will limit ionic and electronic conduction through the film. Additionally, the methyl- and methoxysilyl- 4,4' substituents present after chemisorption of the [Re] precursor to the Si NC surface is expected to shift $E^{o\prime}$ for all features cathodically. Based on literature values, the maximum shift should be no greater than 150 mV due to inductive effects[2] and thus this discussion relies on general trends in the observed features rather than absolute E_p and $E^{o\prime}$ values. That the formal [$Re^{I/0}$] and [$Re^{0/-1}$] features remain consistent with expectations for an untethered [Re] strongly suggests a lack of hybridization with the Si NC band structure. The first CV cycle measured for thin films of B:Si-C_{Ar}[Re] is nearly indistinguishable to the first cycle of B:Si-CH_3CN ($E_{p,c}$ = –1.66 V vs. $Fc^{+/0}$). However, subsequent cycles are quite distinct from both B:Si-CH_3CN and B:Si-OCH_2[Re]. Over 11 cycles the –1.66 V vs. $Fc^{+/0}$ peak in B:Si-C_{Ar}[Re] remains and a broad feature evolves at $E_{p,c}$ ~ –1.94 V vs. $Fc^{+/0}$, resembling the irreversible wave observed for B:Si-CH_3CN on the 11th cycle ($E_{p,c}$ = –1.91 V vs. $Fc^{+/0}$). These results are indicative of strong coupling between the Si NC and surface [Re] complex, where the change in fermi level at the electrode charges the NC film but does not directly alter the oxidation state of [Re].[20]

On anodic scanning of B:Si-CH_3CN films on GCE, there is broad oxidation beginning ca. –1.0 V vs. $Fc^{+/0}$, which depletes to a relatively stable irreversible oxidation ($E_{p,a}$ = 0.05 V vs. $Fc^{+/0}$) on repeat cycling (Fig. S11). This irreversible feature is close to the value expected for the bulk Si VB (ca. 0.4 V vs. NHE, or ca. –0.23 V vs. $Fc^{+/0}$)[21] and may correspond to depopulation of the B:Si VB. We posit the initial broad slope in the anodic current as oxidation of available surface hydrides (*Si-H and *B-H) or reactive silyl (*SiH_3) moieties. On cathodic scanning, there is a slight reductive wave ($E_{p,c}$ = –1.66 V vs. $Fc^{+/0}$) (Fig. 6). However, the broad oxidation observed positive of –1 V vs. $Fc^{+/0}$ induces a large and broad reductive event, peaking at ~ –2 V vs. $Fc^{+/0}$, on the second cycle. After 11 cycles, the broad cathodic feature depletes to a more canonically irreversible wave ($E_{p,c}$ = –1.91 V vs. $Fc^{+/0}$). Again, the cathodic current onset is appropriately negative of the bulk Si CB (ca. –0.7 V vs. NHE, or ca. –1.33 V vs. $Fc^{+/0}$) for the CB of a quantum confined NC and the observed cathodic current may correspond to the population of the B:Si NC CB. Of course, the E_p values for the features described above are not indicative of the actual Si NC band edges as the broad current onsets and general irreversibility of the waves make exact determination difficult.

To better understand the effects of direct tethering in B:Si-CAr[Re] films we undertake a set of precycling experiments. First, we cycle the film between ~ –1.0 to ~0.5 V vs. $Fc^{+/0}$ (before the onset of any cathodic features), we then subject the same film to a series of subsequent CV cycles across the entire potential region (~ 0.5 – –2.5 V vs. $Fc^{+/0}$). This experiment ensures that oxidation induced subspecies have sufficient time to form, stabilizing the cathodic features of the voltammogram. To validate the methodology, we perform this experiment on the same samples described above and observe the same trends in the voltametric responses over multiple cycles (Fig. S11).

To verify our findings are a result of direct tethering we, thin films of B:Si+[Re]N_2 are subjected to the oxidative precycling experiment described above (Fig. S12). The resulting cathodic scan after oxidative treatment of the film closely matches the corresponding B:Si-OCH_2[Re] film, with three features present in the –1.4 – –2.4 V vs. $Fc^{+/0}$ range ($E_{p,c}$ = –1.67, –1.78, and –2.17 V vs. $Fc^{+/0}$, $\Delta E_{p2,p3}$ = 0.39). Conversely, films of B:Si-C_{Ar}[Re] subjected to the same treatment only show the initial B:Si related peak ($E_{p,c}$ = –1.65 V vs. $Fc^{+/0}$) described above.

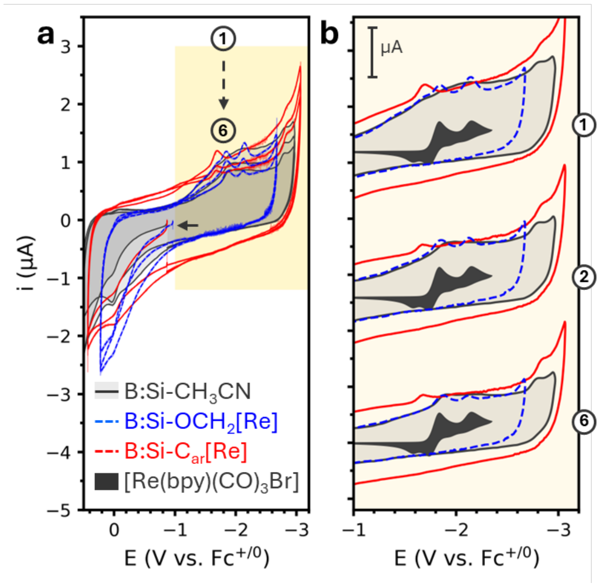

Fig. S12 (a) CVs of B:Si-CH_3CN (black line, grey shading), B:Si-OCH_2[Re] (blue dashed trace), and B:Si-C_{Ar}[Re] (red solid line) thin films on glassy carbon. The first, second, and sixth cycles are shown and indicated by the circled numbers. A voltammogram of 1 mM [Re(bpy)$(CO)_3$Br] (black shaded trace) is included for comparison and scaled to 1/12th current for clarity. (b) Derivative voltammograms for the CV data shown in (a). All voltammograms are collected at 50 mV·s^{-1} in CH_3CN under an argon atmosphere.

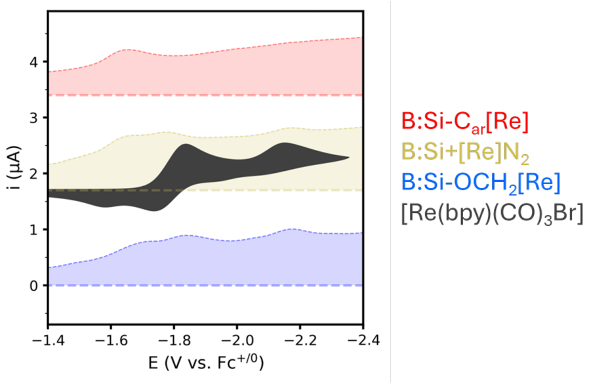

Fig. S13 Linear scan voltammograms of thin films of B:Si-C_{Ar}[Re] (red), B:Si+[Re]N_2 (yellow), and B:Si-OCH_2[Re] (blue). A scaled (by 1/13th) CV of dissolved [Re(bpy)$(CO)_3$Br] (black) is included for comparison. Voltammograms are offset for clarity with the thick dashed lines representing 0 for each distinct trace.

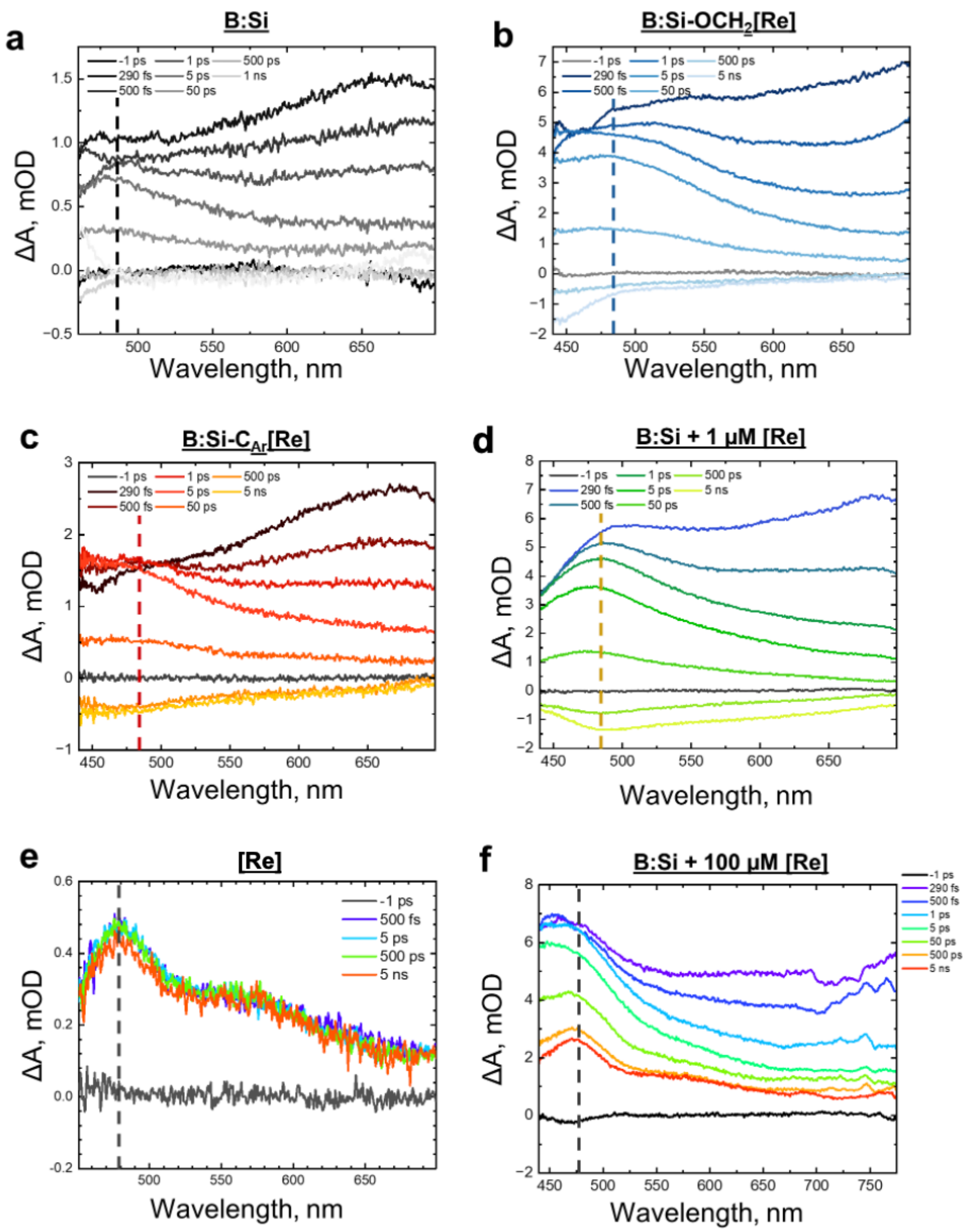

Fig. S14 Transient absorption spectra recorded after excitation at 400 nm of a) 1 µM B:Si-CH_3CN, b) 1 µM B:Si-OCH_2[Re], c) 1 µM B:Si-C_{Ar}[Re], d) a mixture of 1 µM B:Si-CH_3CN and 1 µM [Re(bpy)$(CO)_3$Br], e) 500 µM [Re(bpy)$(CO)_3$Br], and f) a mixture of 1 µM B:Si-CH_3CN and 1 µM [Re(bpy)$(CO)_3$Br]. 3.9 nm ^{i}Si-C_{12}. All spectra acquired after excitation at 400 nm, samples were prepared at 1 µM in a 1:4 mixture of toluene:THF under an Ar atmosphere. All solutions prepared in CH_3CN under an Ar atmosphere.

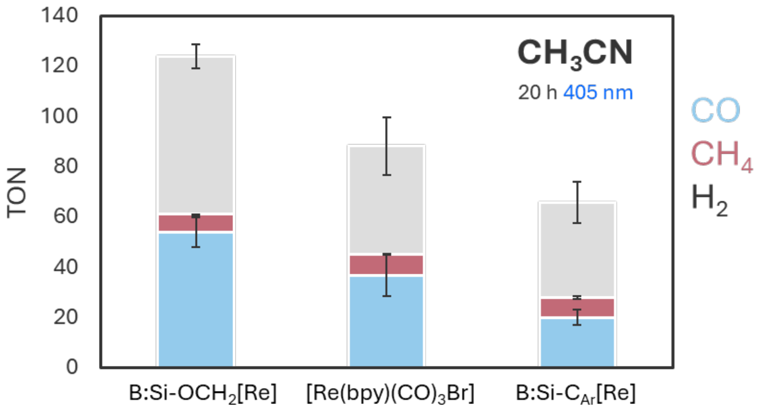

Fig. S15 Photocatalytic formation of H_2 (grey), CH_4 (red), and CO (blue) by B:Si-OCH$_2$[Re], [Re(bpy)(CO)$_3$Br], and B:Si-C$_{Ar}$[Re]. Error bars representing the standard deviation between three separate photocatalytic measurements are included.

References

1. Saund, S. S.; Dabak-Wakankar, A.; Gish, M. K.; Neale, N. R., Silicon nanocrystal hybrid photocatalysts as models to understand solar fuels producing assemblies. *Sustain. Energ. Fuels* **2024,** *8*, 403-409.
2. Smieja, J. M.; Kubiak, C. P., Re(bipy-tBu)(CO)3Cl−improved Catalytic Activity for Reduction of Carbon Dioxide: IR-Spectroelectrochemical and Mechanistic Studies. *Inorg. Chem.* **2010,** *49*, 9283-9289.
3. Carroll, G. M.; Limpens, R.; Neale, N. R., Tuning Confinement in Colloidal Silicon Nanocrystals with Saturated Surface Ligands. *Nano Lett.* **2018,** *18*, 3118-3124.
4. Limpens, R.; Pach, G. F.; Mulder, D. W.; Neale, N. R., Size-Dependent Asymmetric Auger Interactions in Plasma-Produced n- and p-Type-Doped Silicon Nanocrystals. *J. Phys. Chem. C* **2019,** *123*, 5782-5789.
5. Wheeler, L. M.; Anderson, N. C.; Palomaki, P. K. B.; Blackburn, J. L.; Johnson, J. C.; Neale, N. R., Silyl Radical Abstraction in the Functionalization of Plasma-Synthesized Silicon Nanocrystals. *Chem. Mater.* **2015,** *27*, 6869-6878.
6. Choate, J. C.; Silva, I., Jr.; Hsu, P. C.; Tran, K.; Marinescu, S. C., The Positional Effect of an Immobilized Re Tricarbonyl Catalyst for CO2 Reduction. *ACS Appl. Mater. Interfaces* **2024,** *16*, 50534-50549.
7. Schulze, M. C.; Carroll, G. M.; Martin, T. R.; Sanchez-Rivera, K.; Urias, F.; Neale, N. R., Hydrophobic versus Hydrophilic Interfacial Coatings on Silicon Nanoparticles Teach Us How to Design the Solid Electrolyte Interphase in Silicon-Based Li-Ion Battery Anodes. *ACS Appl. Energy Mater.* **2021,** *4*, 1628-1636.
8. Neese, F., The ORCA program system. *Wiley Interdiscip. Rev. Comput. Mol. Sci.* **2012,** *2*, 73-78.
9. Neese, F., Software update: the ORCA program system, version 4.0. *Wiley Interdiscip. Rev. Comput. Mol. Sci.* **2018,** *8*, e1327.
10. Neese, F.; Wennmohs, F.; Becker, U.; Riplinger, C., The ORCA quantum chemistry program package. *J. Chem. Phys.* **2020,** *152*, 224108.
11. Valeev, E. F. *Libint: A library for the evaluation of molecular integrals of many-body operators over Gaussian functions*, 2.9.0; http://libint.valeyev.net/, 2024.
12. Lehtola, S.; Steigemann, C.; Oliveira, M. J. T.; Marques, M. A. L., Recent developments in libxc — A comprehensive library of functionals for density functional theory. *SoftwareX* **2018,** *7*, 1-5.

13. Ekström, U.; Visscher, L.; Bast, R.; Thorvaldsen, A. J.; Ruud, K., Arbitrary-Order Density Functional Response Theory from Automatic Differentiation. *J. Chem. Theory Comput.* **2010,** *6*, 1971-1980.
14. Weigend, F.; Ahlrichs, R., Balanced basis sets of split valence, triple zeta valence and quadruple zeta valence quality for H to Rn: Design and assessment of accuracy. *Phys. Chem. Chem. Phys.* **2005,** *7*, 3297-3305.
15. Pantazis, D. A.; Chen, X.-Y.; Landis, C. R.; Neese, F., All-Electron Scalar Relativistic Basis Sets for Third-Row Transition Metal Atoms. *J. Chem. Theory Comput.* **2008,** *4*, 908-919.
16. Weigend, F., Accurate Coulomb-fitting basis sets for H to Rn. *Phys. Chem. Chem. Phys.* **2006,** *8*, 1057-1065.
17. Grimme, S.; Antony, J.; Ehrlich, S.; Krieg, H., A consistent and accurate ab initio parametrization of density functional dispersion correction (DFT-D) for the 94 elements H-Pu. *J. Chem. Phys.* **2010,** *132*, 154104.
18. Kayanuma, Y., Quantum-size effects of interacting electrons and holes in semiconductor microcrystals with spherical shape. *Phys. Rev. B* **1988,** *38*, 9797-9805.
19. Delerue, C.; Allan, G.; Lannoo, M., Theoretical aspects of the luminescence of porous silicon. *Phys. Rev. B: Condens. Matter* **1993,** *48*, 11024-11036.
20. Jackson, M. N.; Oh, S.; Kaminsky, C. J.; Chu, S. B.; Zhang, G.; Miller, J. T.; Surendranath, Y., Strong Electronic Coupling of Molecular Sites to Graphitic Electrodes via Pyrazine Conjugation. *J. Am. Chem. Soc.* **2018,** *140*, 1004-1010.
21. Rehm, J. M.; McLendon, G. L.; Fauchet, P. M., Conduction and Valence Band Edges of Porous Silicon Determined by Electron Transfer. *J. Am. Chem. Soc.* **1996,** *118*, 4490-4491.
